# Supplementary material for: Allosteric zinc inhibition and interdomain regulation govern the catalytic mechanism of the E3-independent ubiquitin-conjugating enzyme hUBE2O
Source: J Biol Chem. 2025 Dec 30;302(2):111122. doi: 10.1016/j.jbc.2025.111122 (PMC12835413; doi:10.1016/j.jbc.2025.111122)

SourceDataF1

F1A

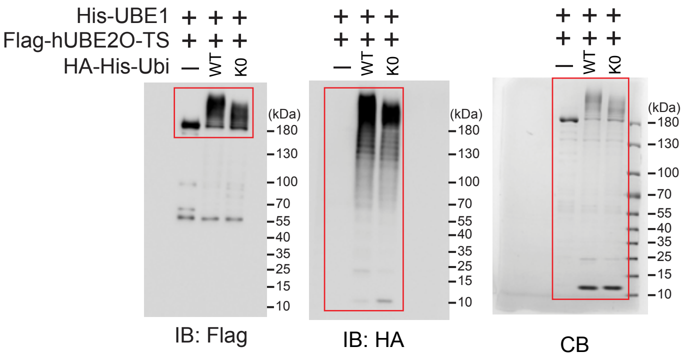

F1E

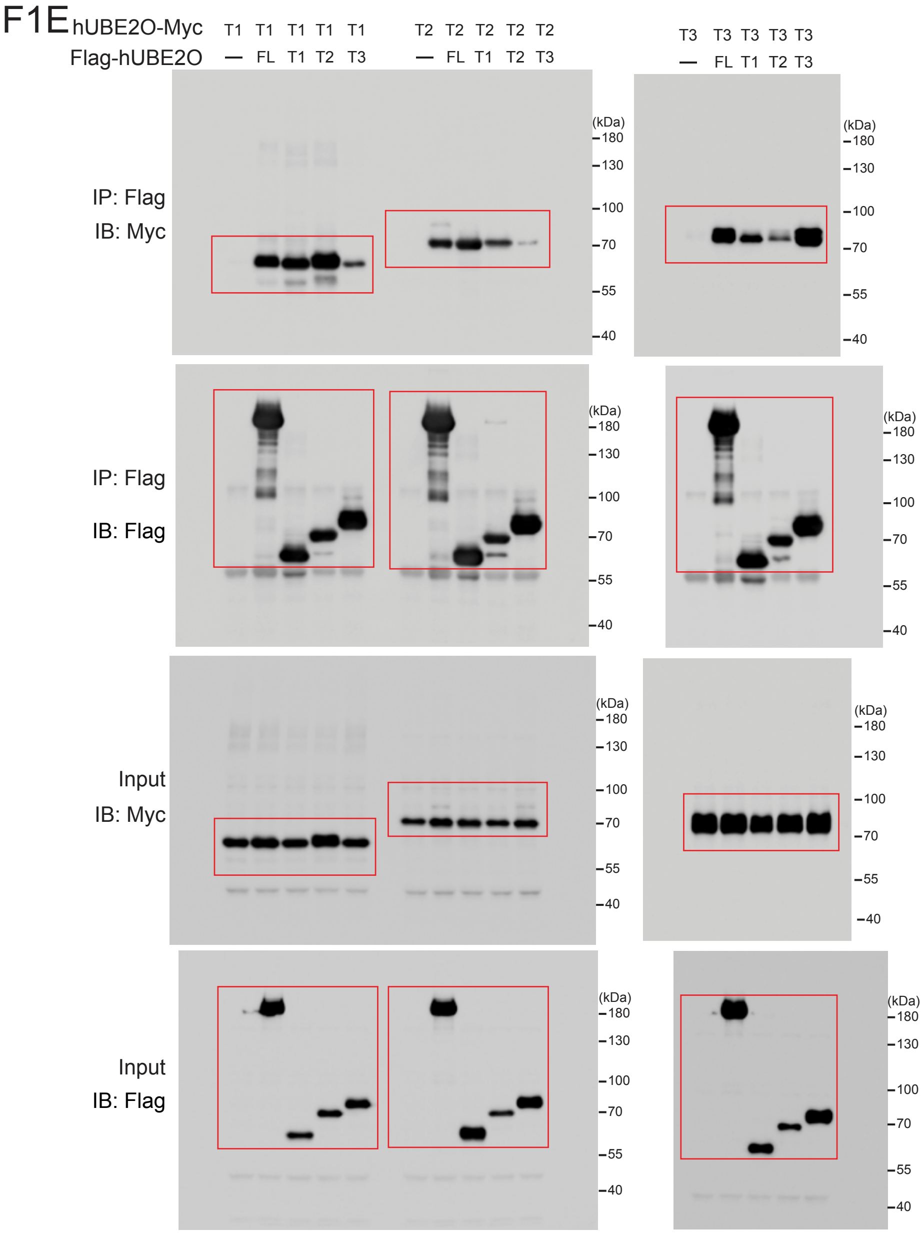

SourceDataF2

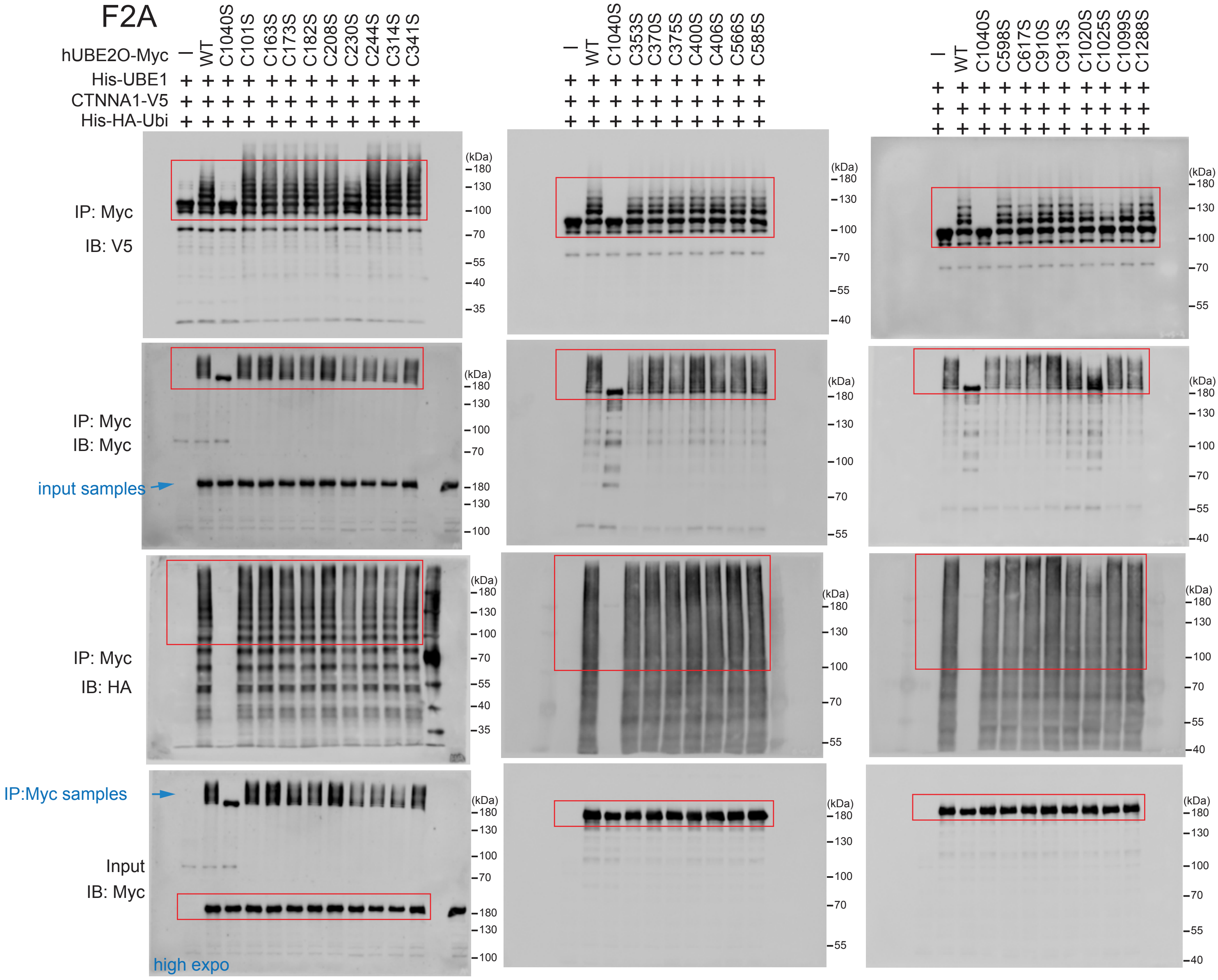

The input and IP samples were resolved
in separate SDS-PAGE gels, then transferred
onto the same nitrocellulose membrane for subsequent
anti-Myc immunoblotting

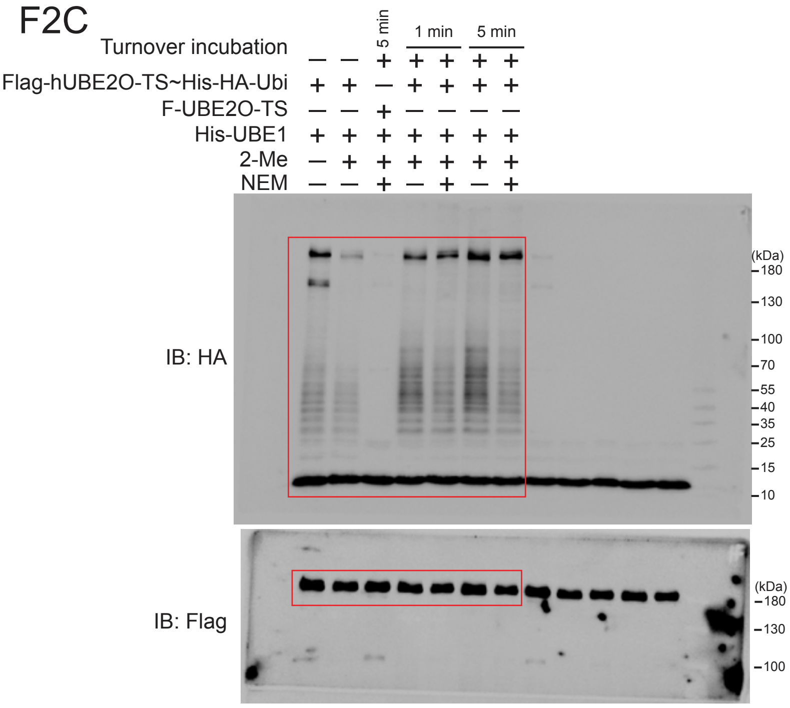

SourceDataF3

F3B

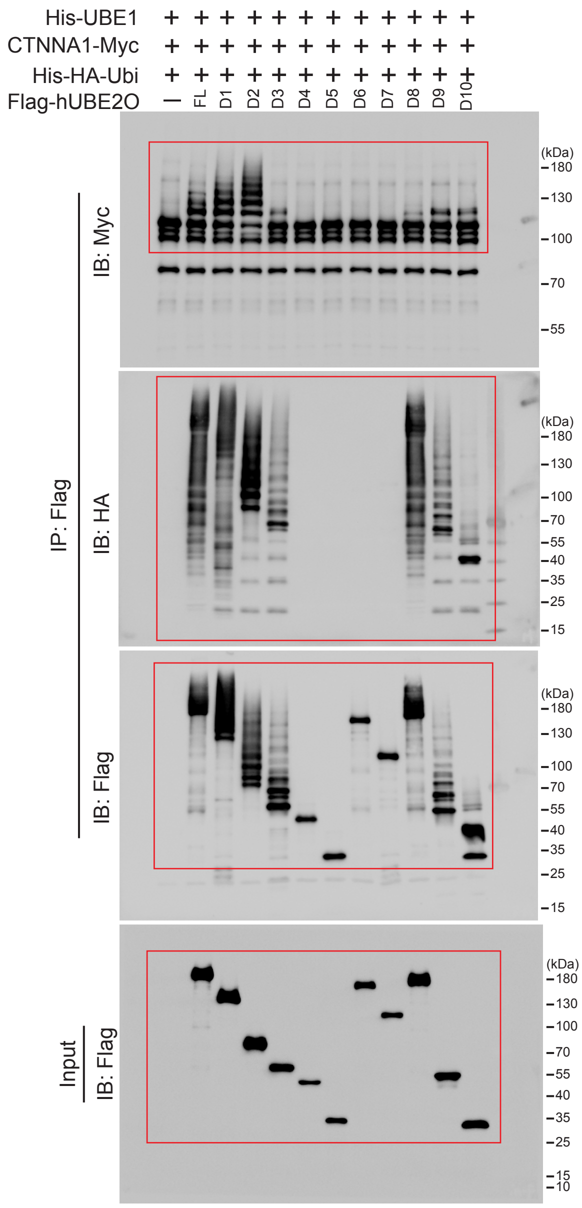

F3D

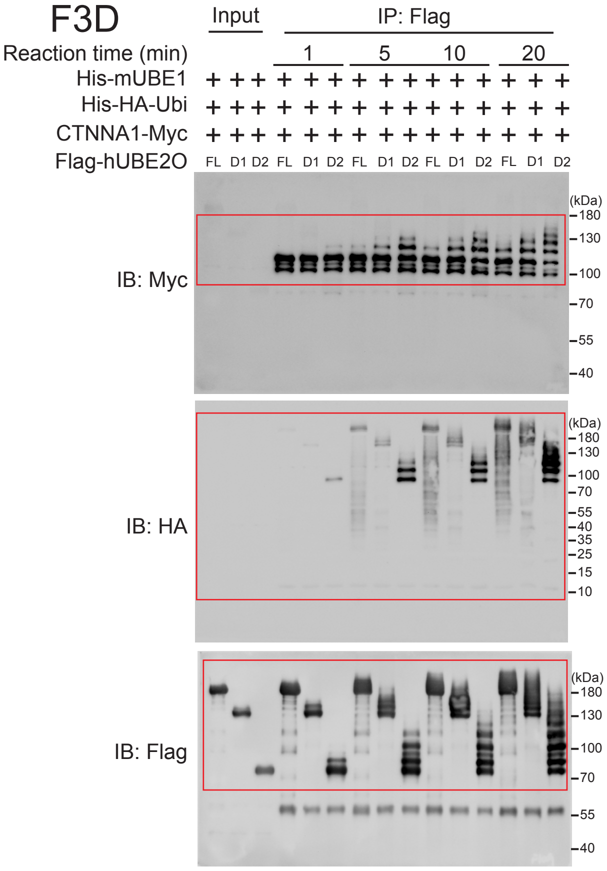

F3C

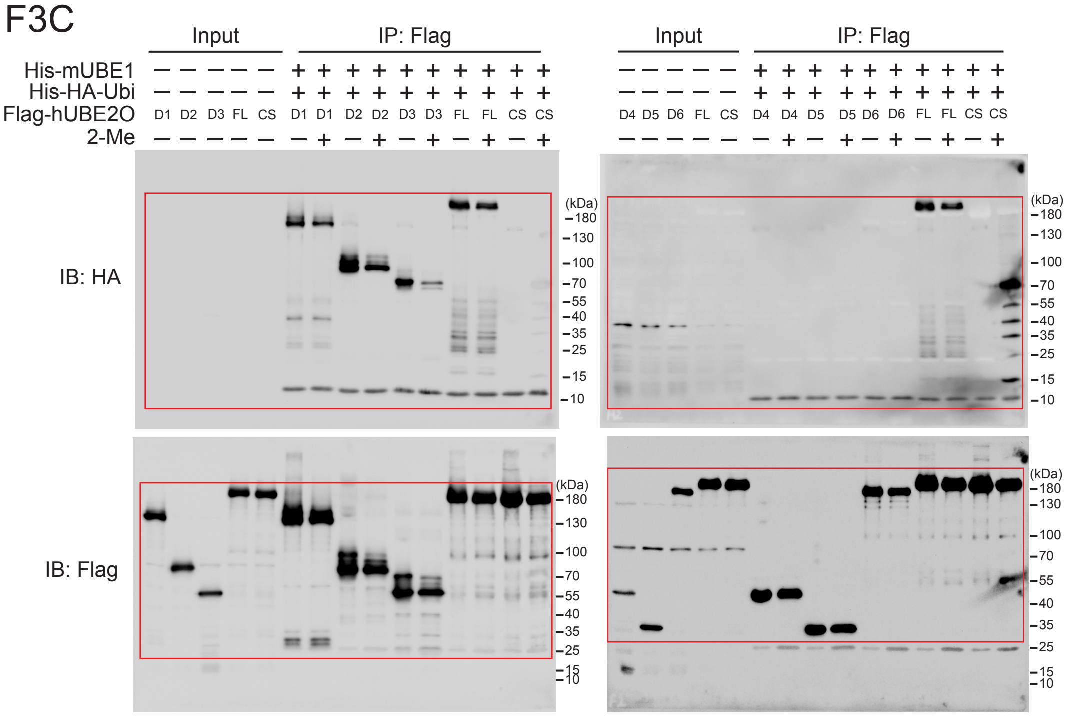

SourceDataF4

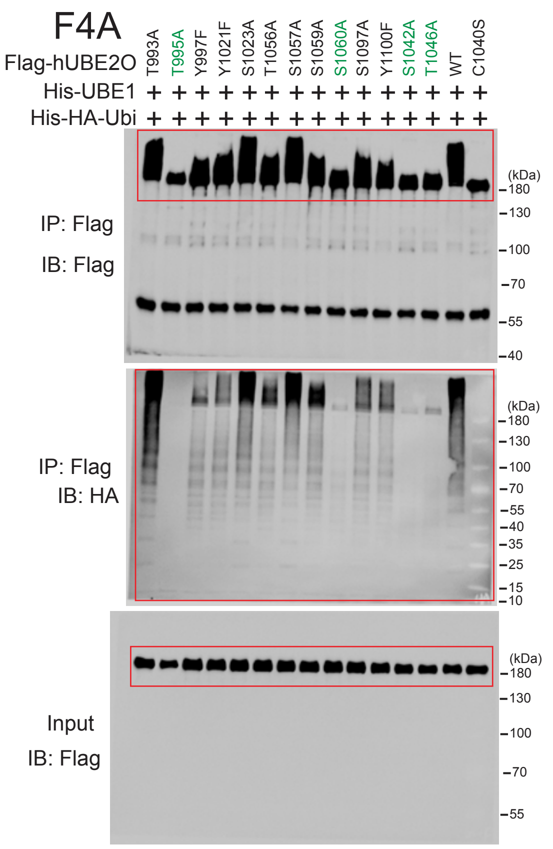

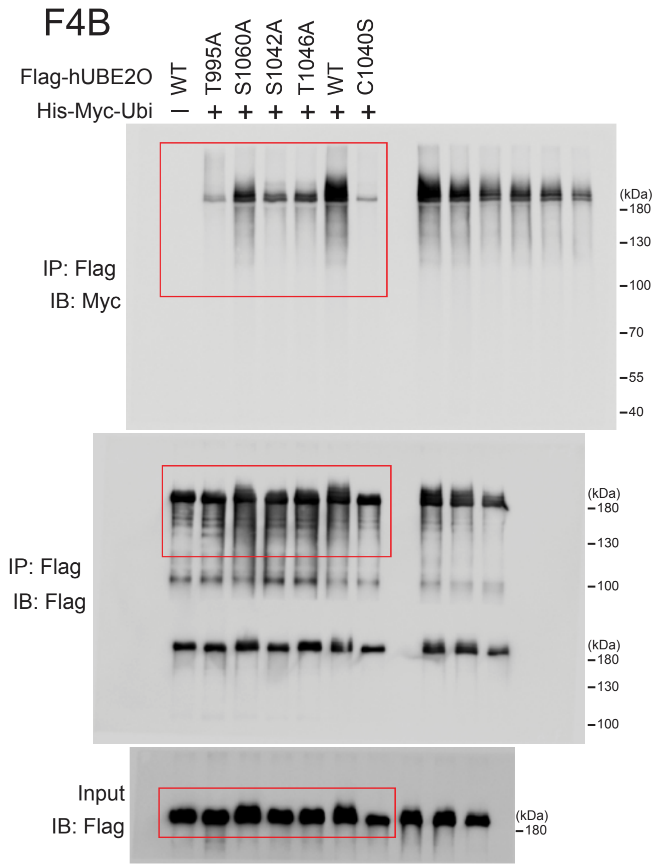

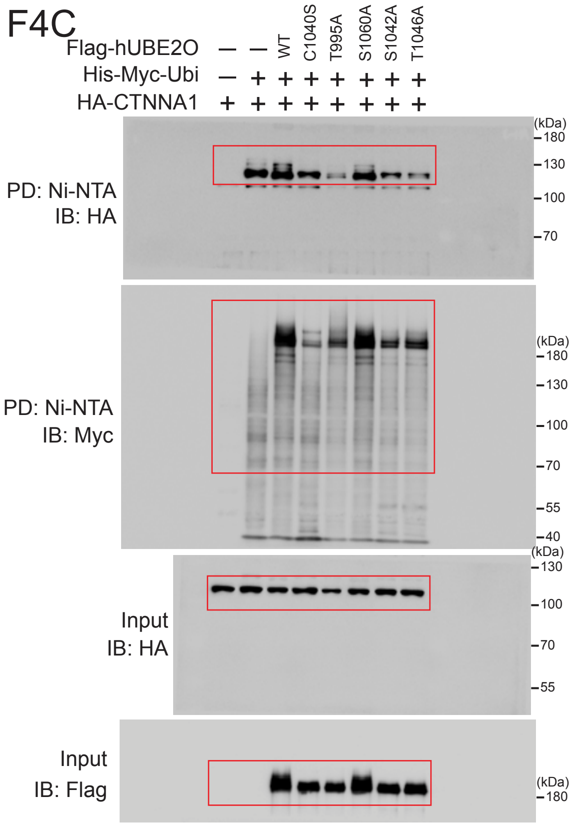

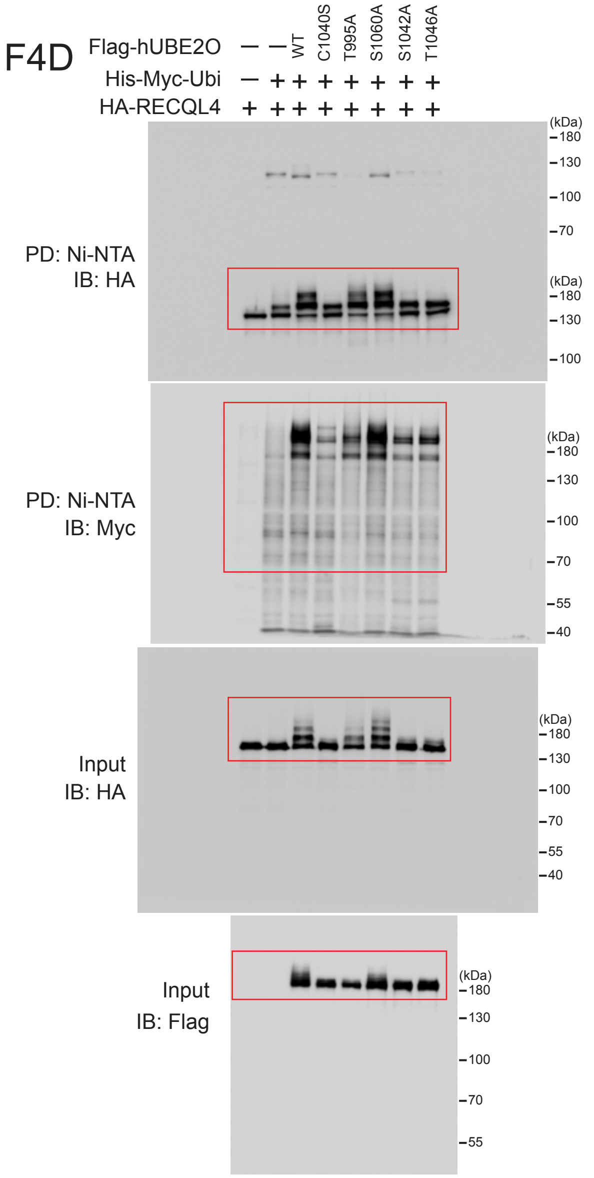

SourceDataF4

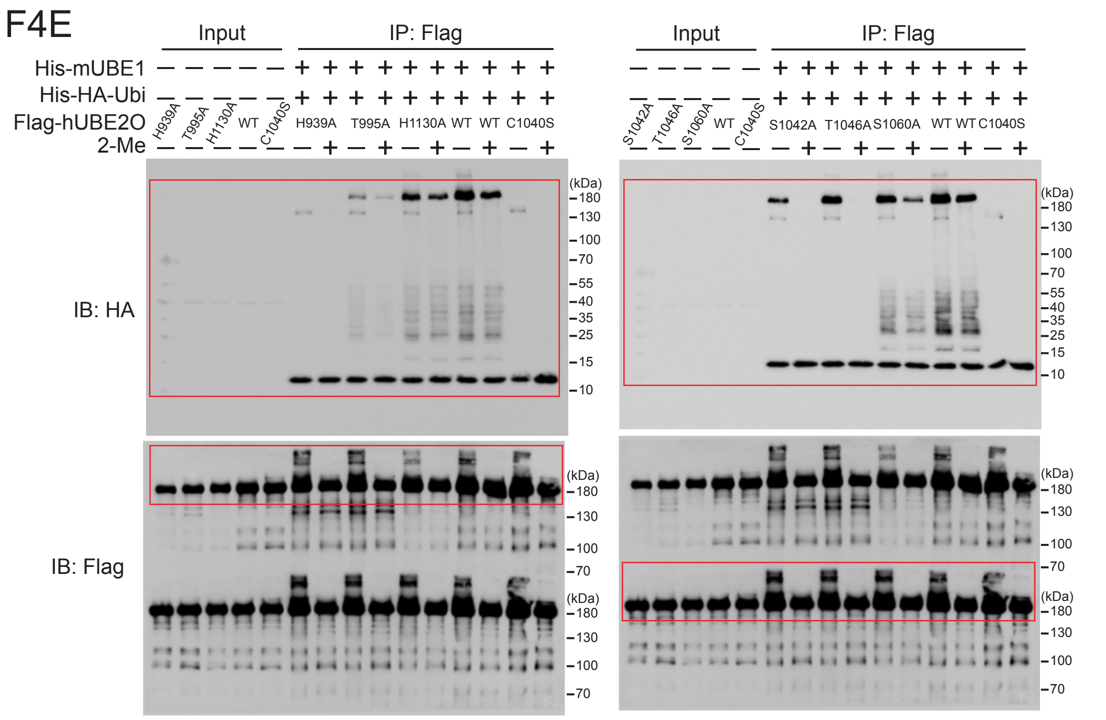

These samples were resolved in separate SDS-PAGE gels, then transferred onto the same nitrocellulose membrane for subsequent anti-Flag immunoblotting

SourceDataF5

F5A

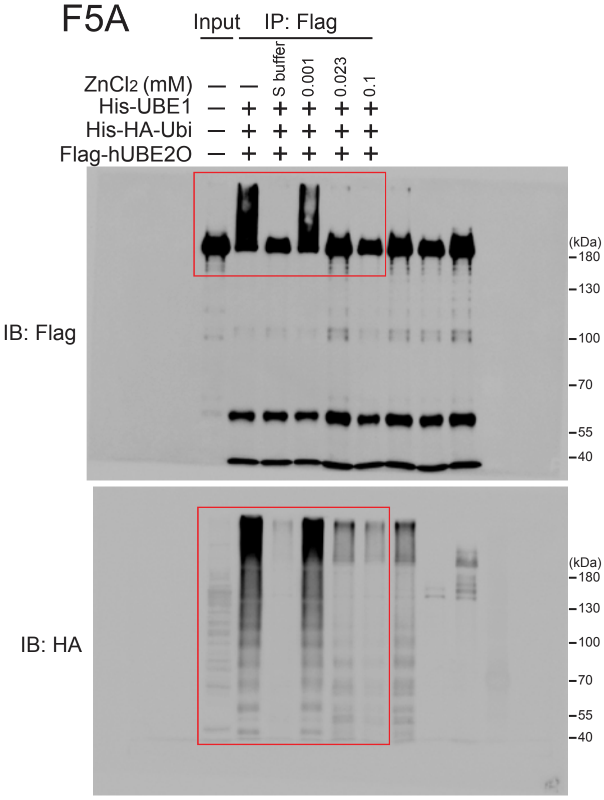

F5C

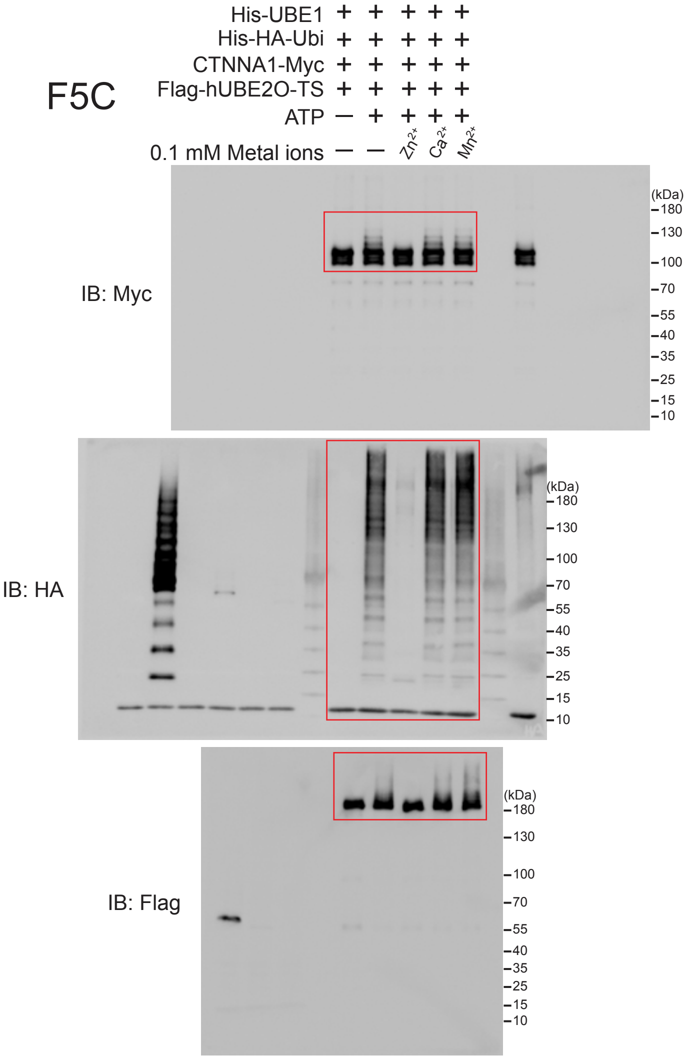

F5D

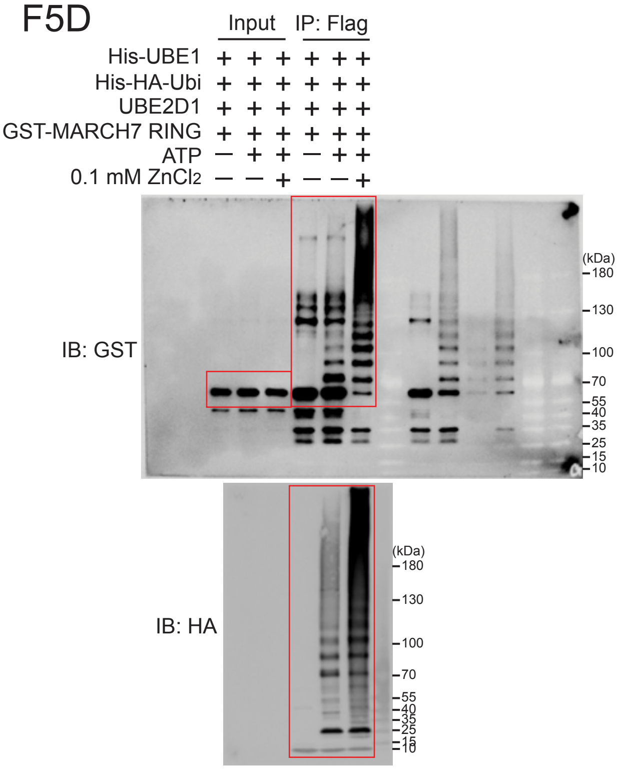

F5E

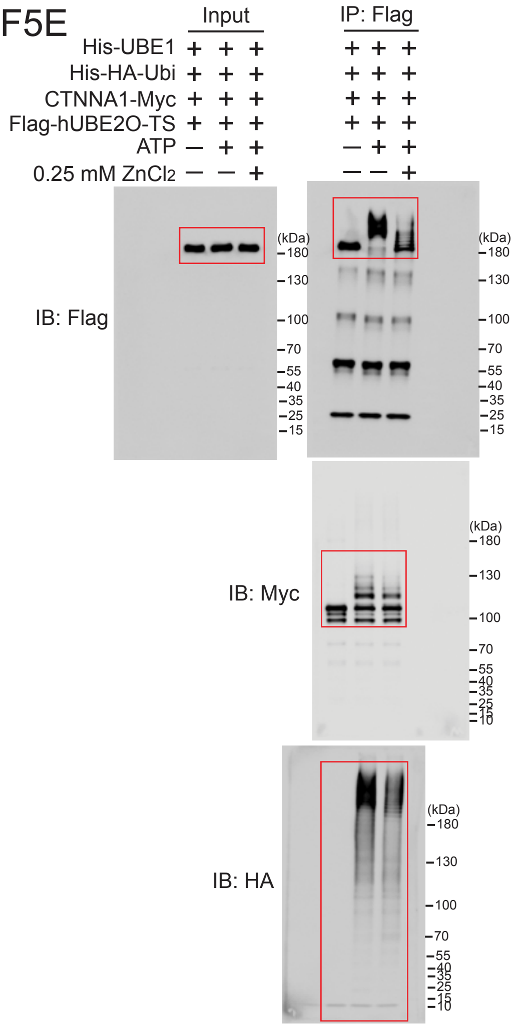

SourceDataF5

F5F

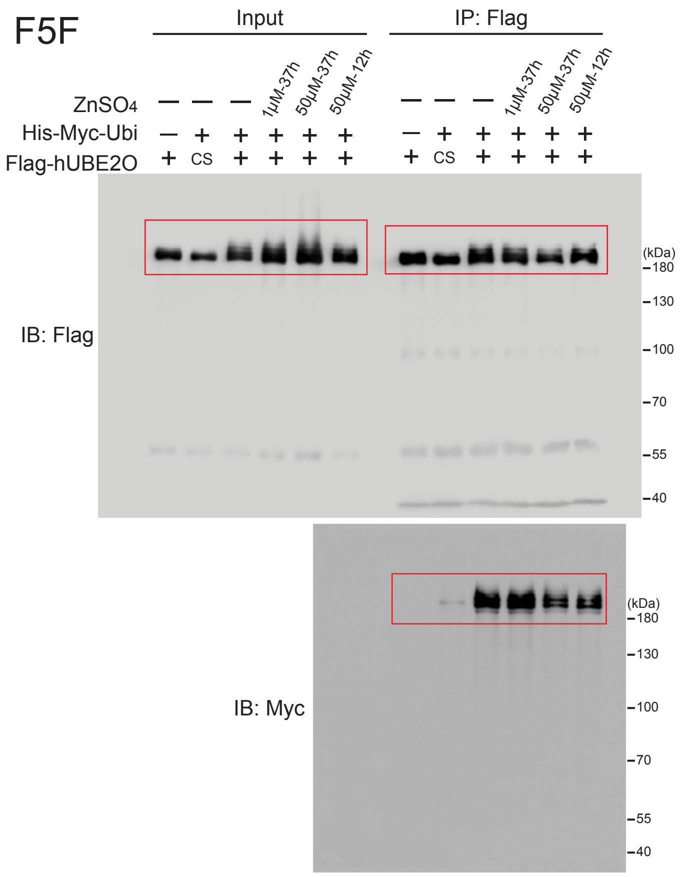

SourceDataF6

F6A

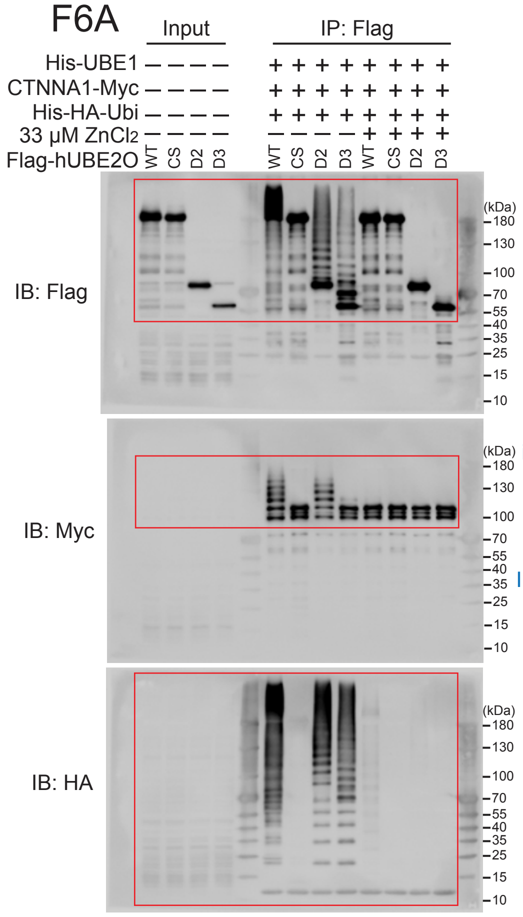

F6B

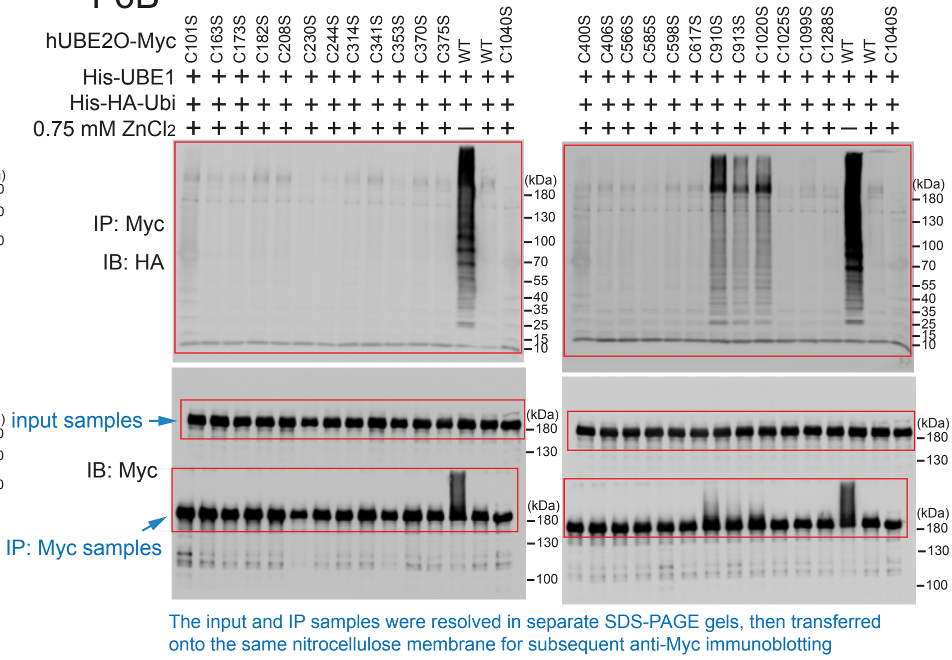

F6D

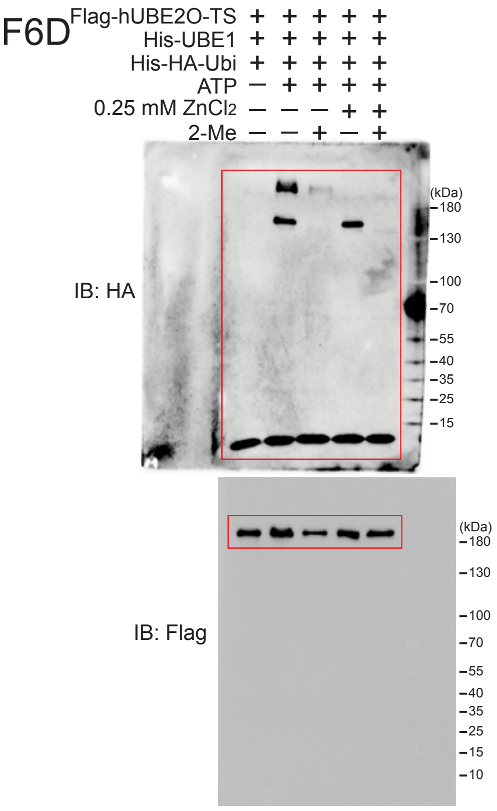

SourceDataFS1

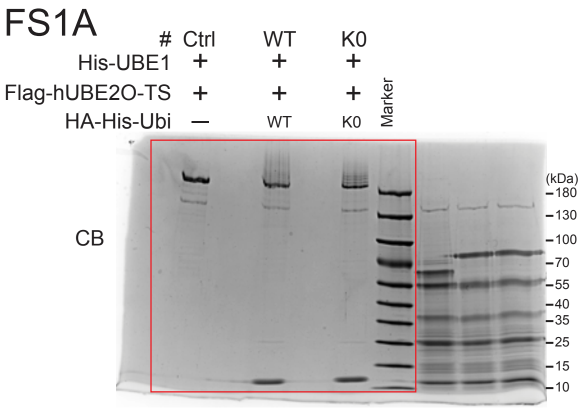

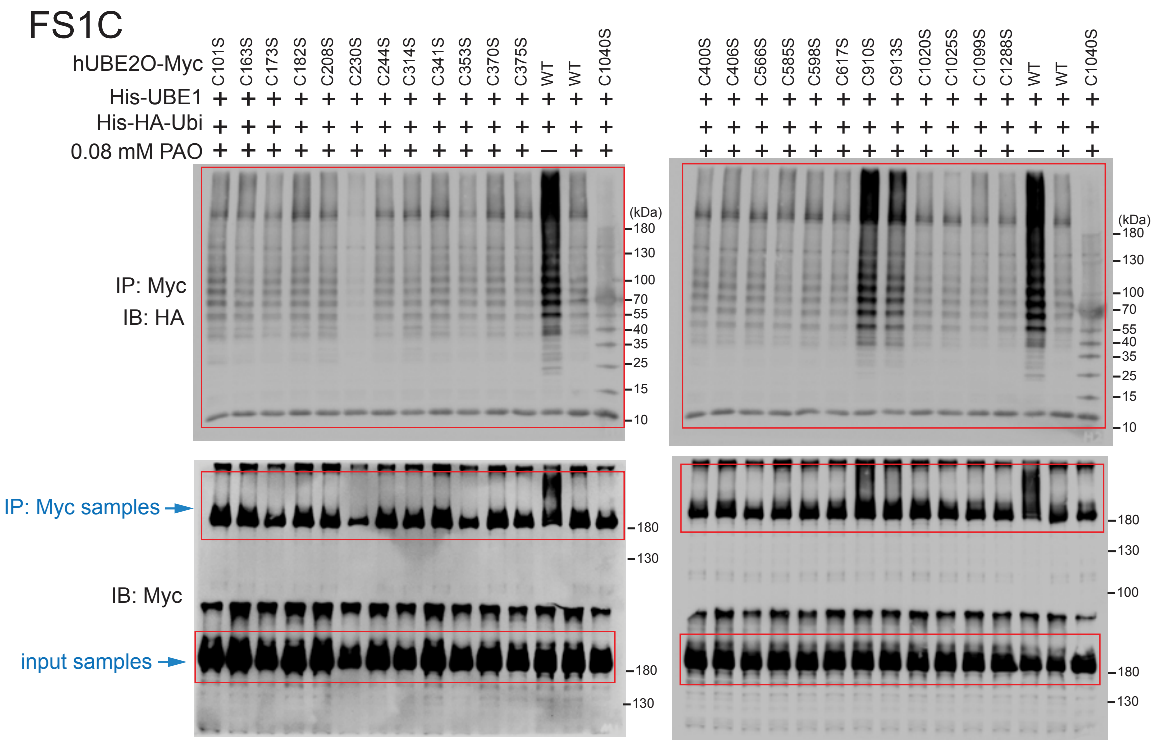

The input and IP samples were resolved in separate SDS-PAGE gels, then transferred onto the same nitrocellulose membrane for subsequent anti-Myc immunoblotting

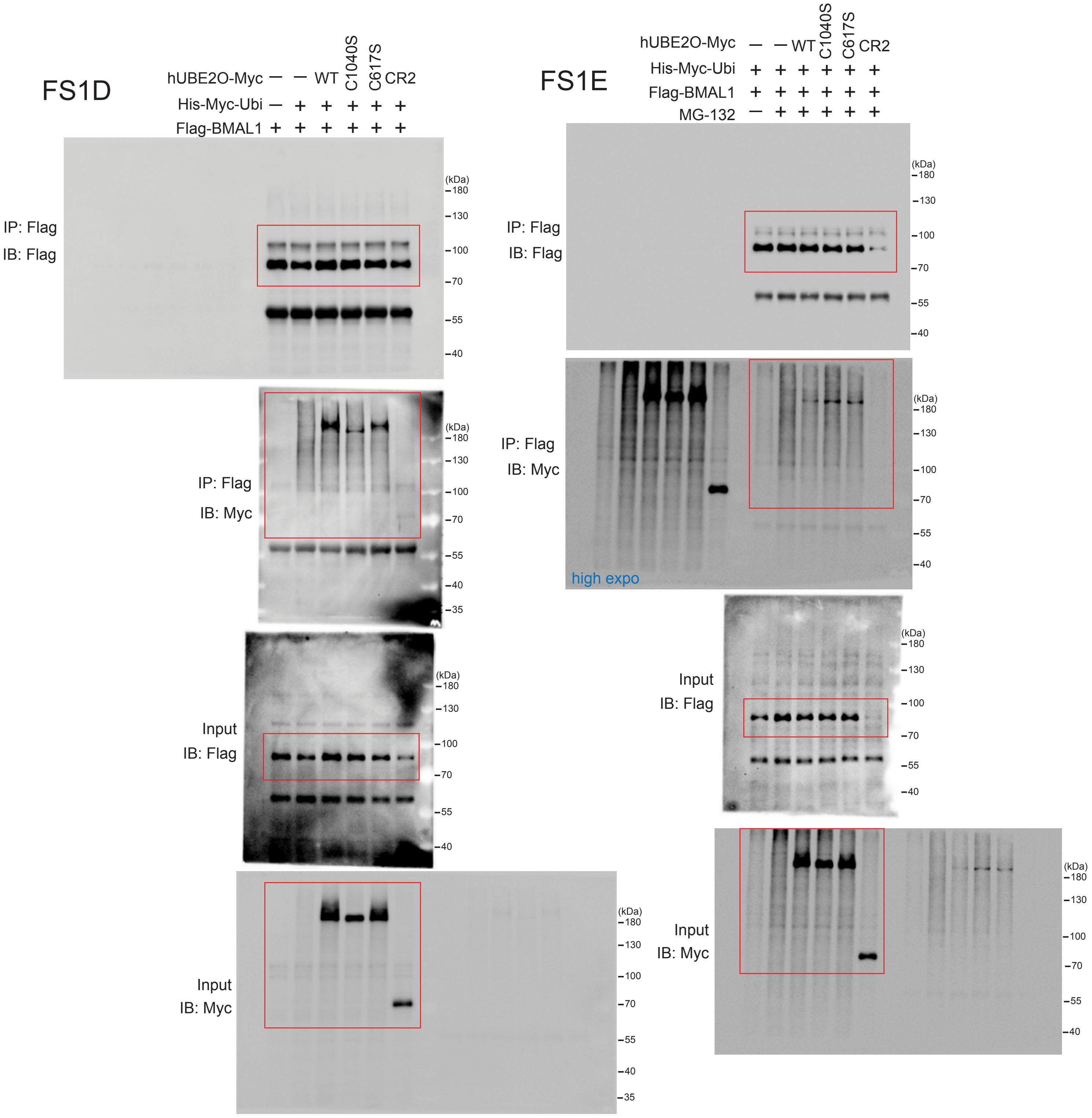
FS1D
hUBE2O-Myc — — WT C1040S C617S CR2
His-Myc-Ubi — + + + + +
Flag-BMAL1 + + + + + +
IP: Flag
IB: Flag
IP: Flag
IB: Myc
Input
IB: Flag
Input
IB: Myc
(kDa)
180
130
100
70
55
40
35
FS1E
hUBE2O-Myc — — WT C1040S C617S CR2
His-Myc-Ubi + + + + + +
Flag-BMAL1 + + + + + +
MG-132 — + + + + +
IP: Flag
IB: Flag
IP: Flag
IB: Myc
high expo
Input
IB: Flag
Input
IB: Myc

SourceDataFS2

FS2B

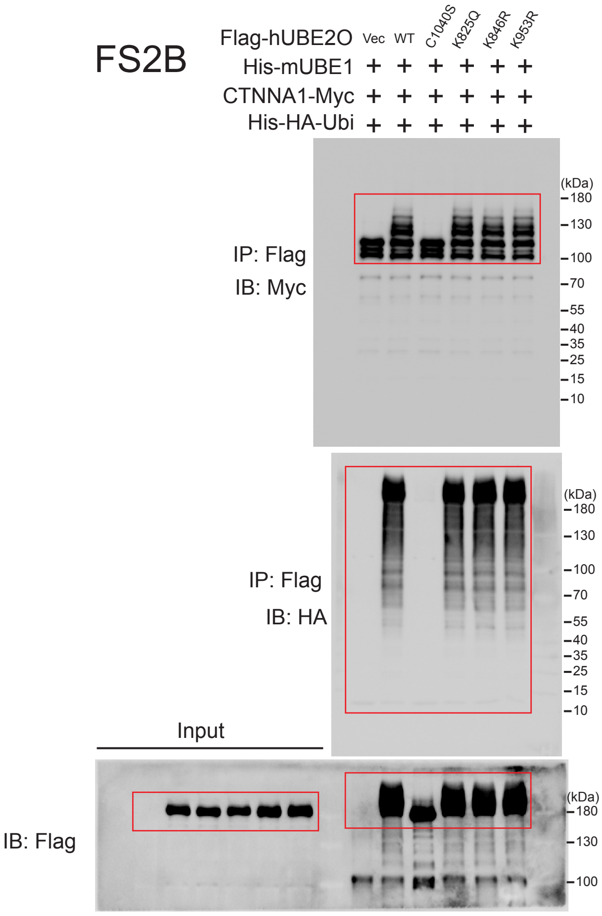

FS2C

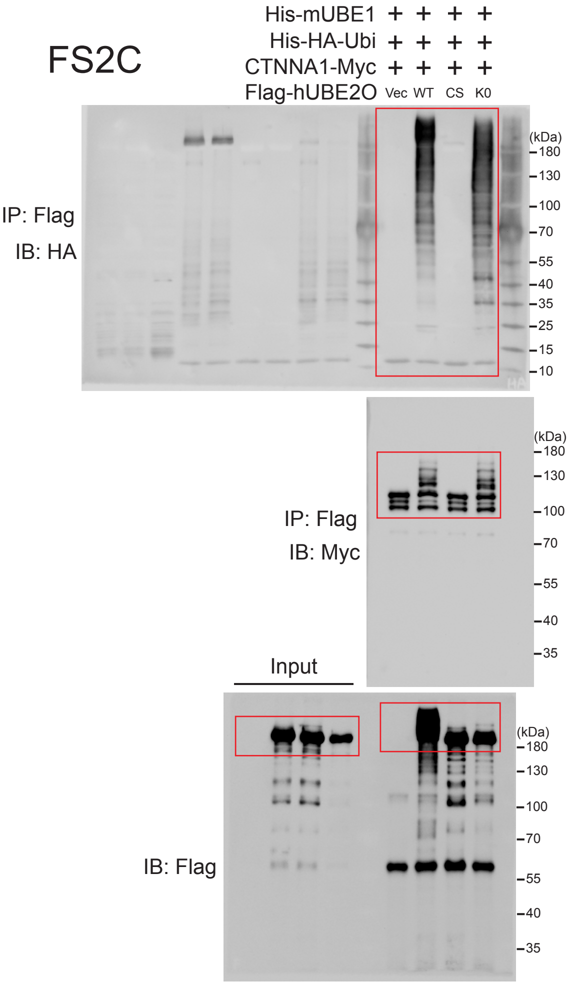

FS2D

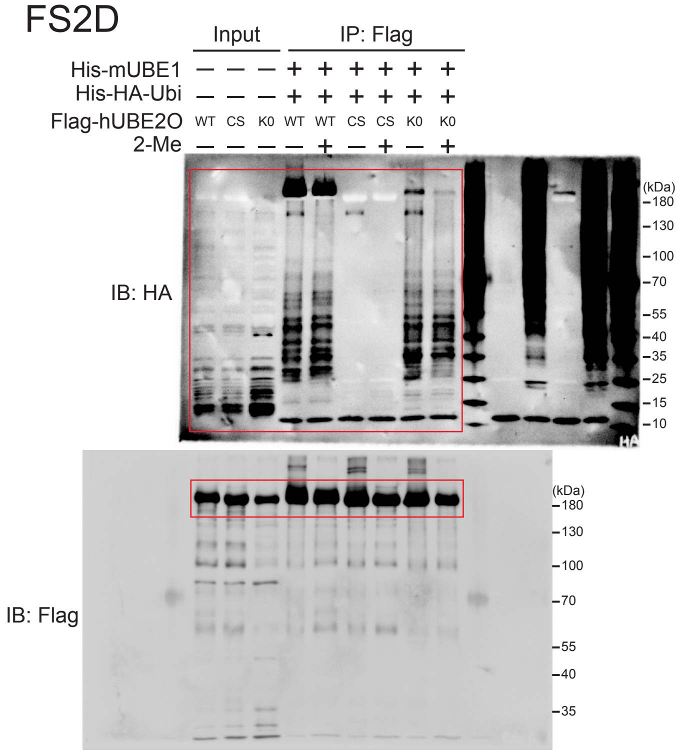

FS2E

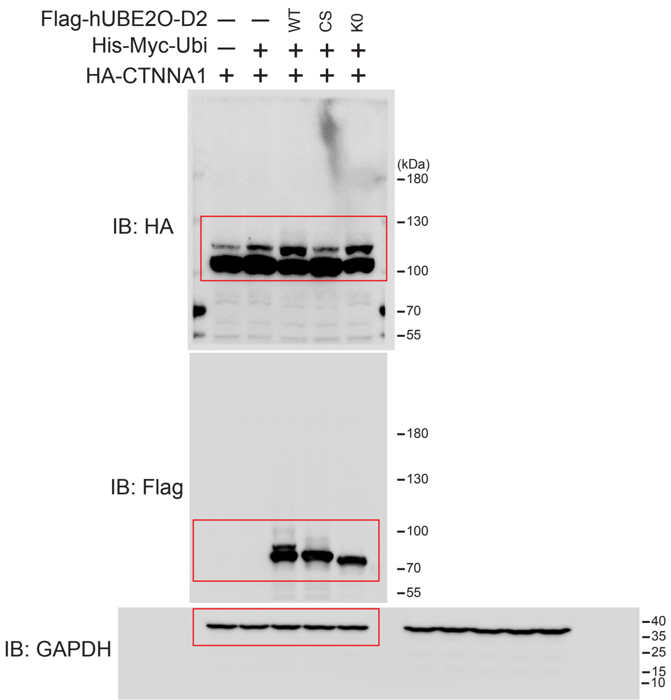

SourceDataFS2

FS2F

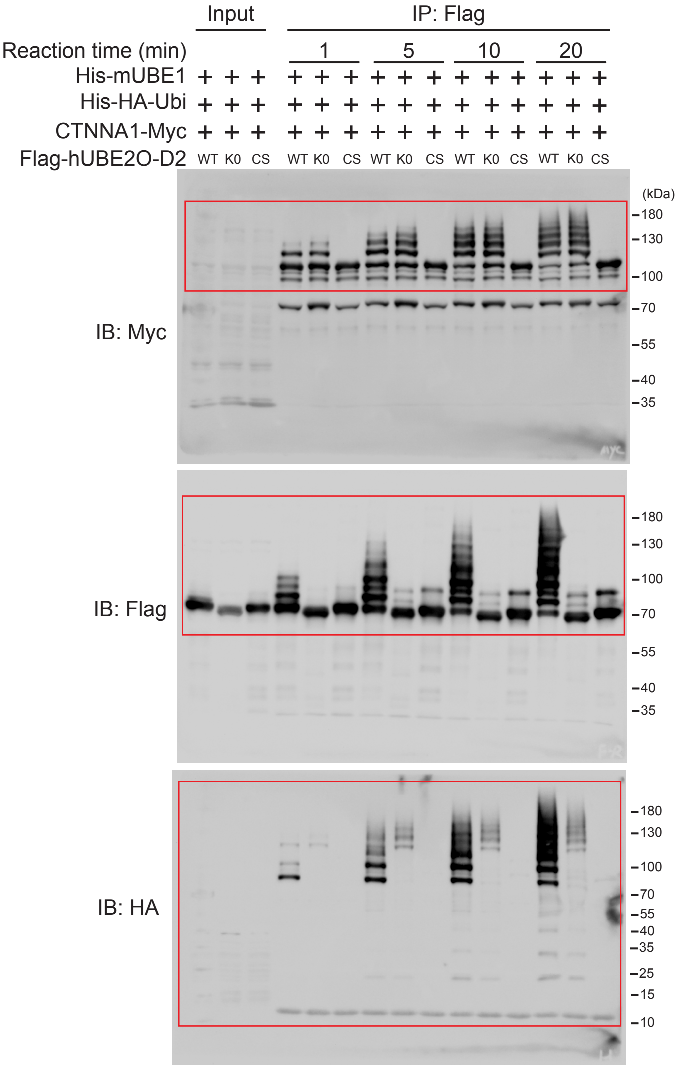

SourceDataFS3

FS3A

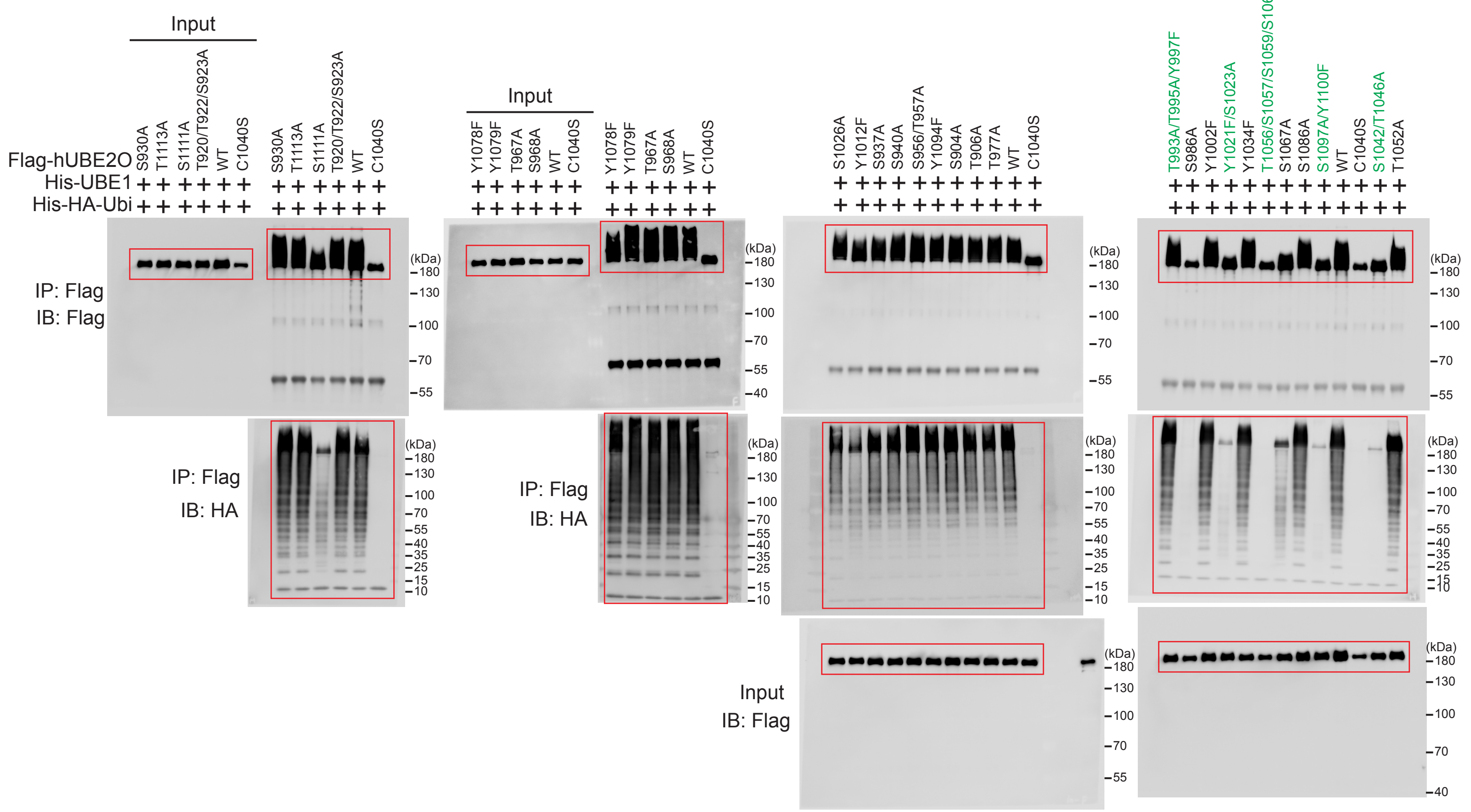

FS3B

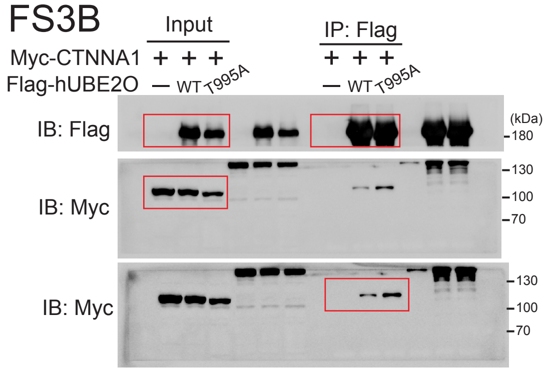

FS3C

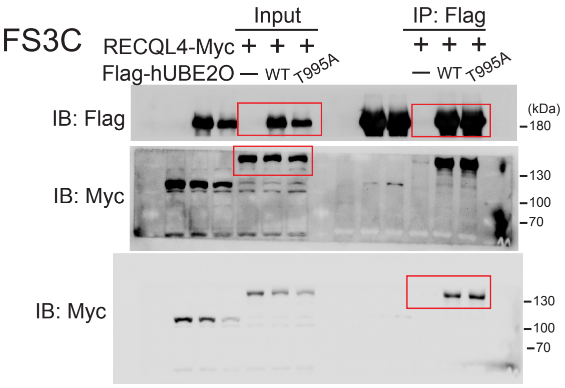

FS3D

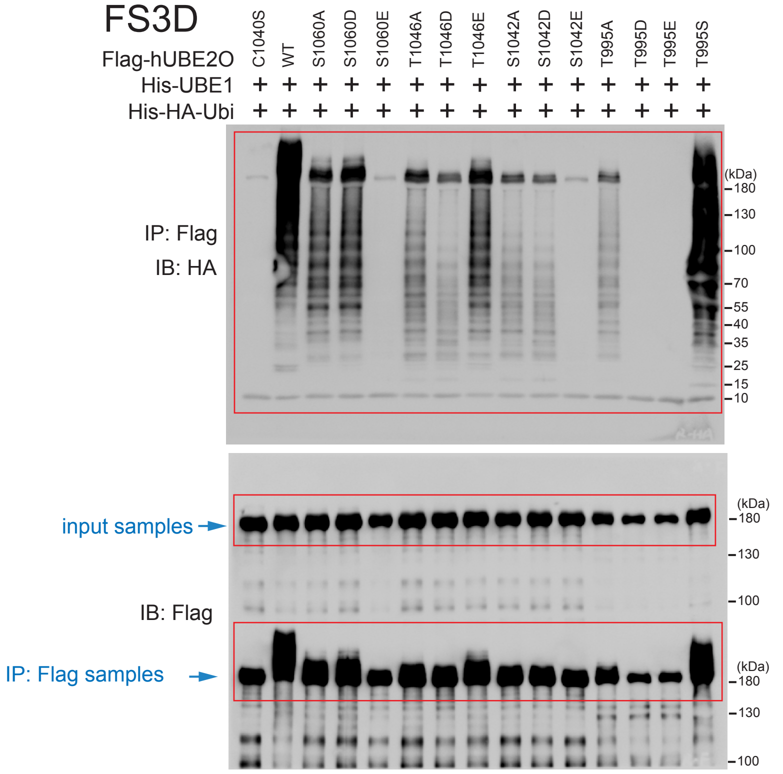

The input and IP samples were resolved
in separate SDS-PAGE gels, then transferred
onto the same nitrocellulose membrane for subsequent
anti-Flag immunoblotting

SourceDataFS4

FS4A

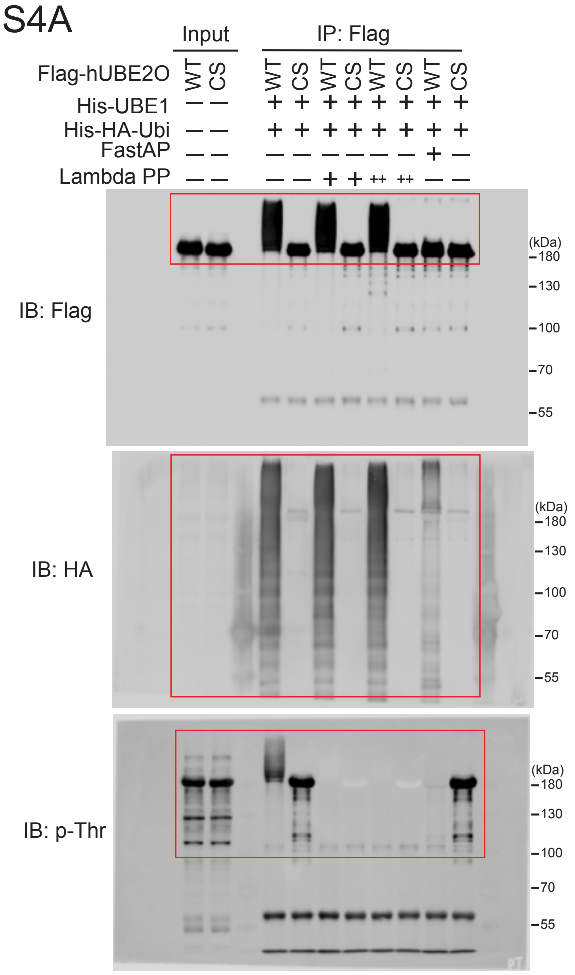

FS4B

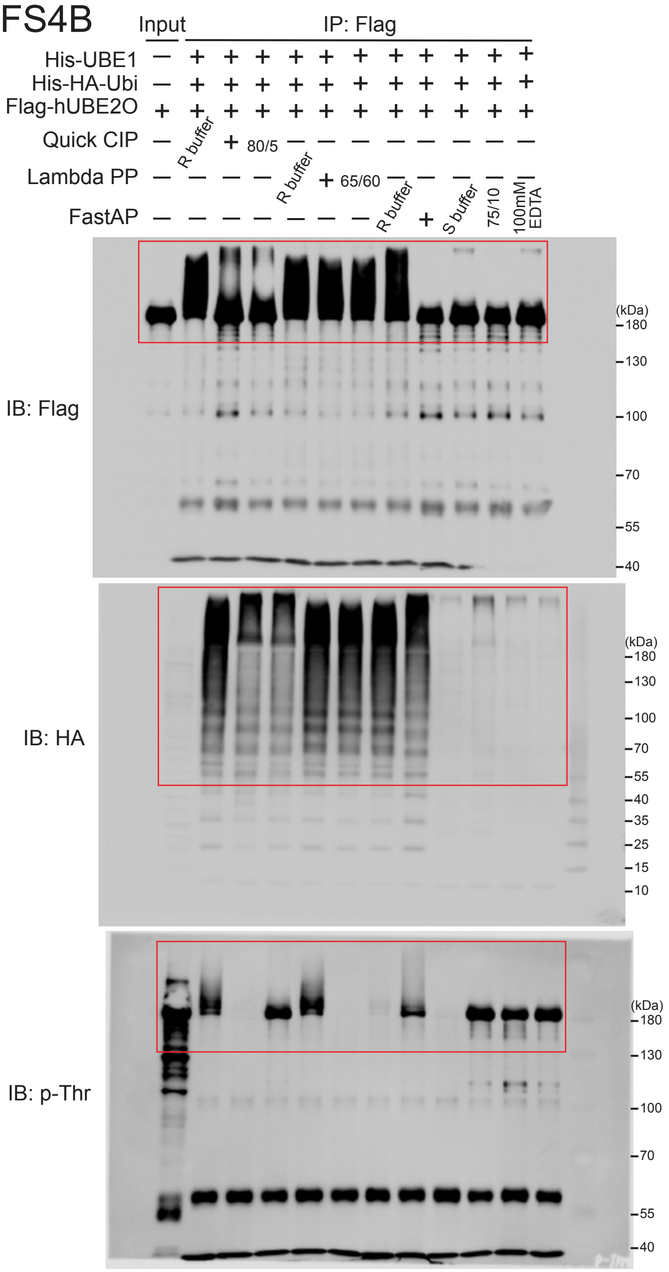

FS4C

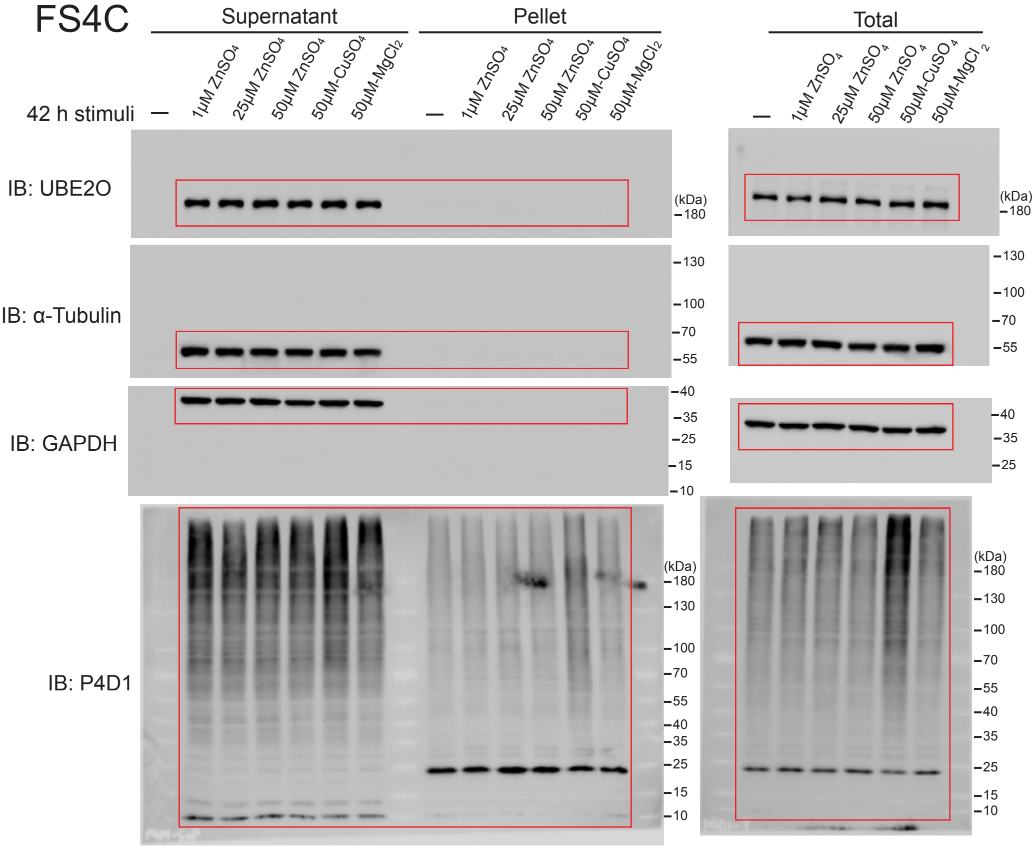

SourceDataFS5

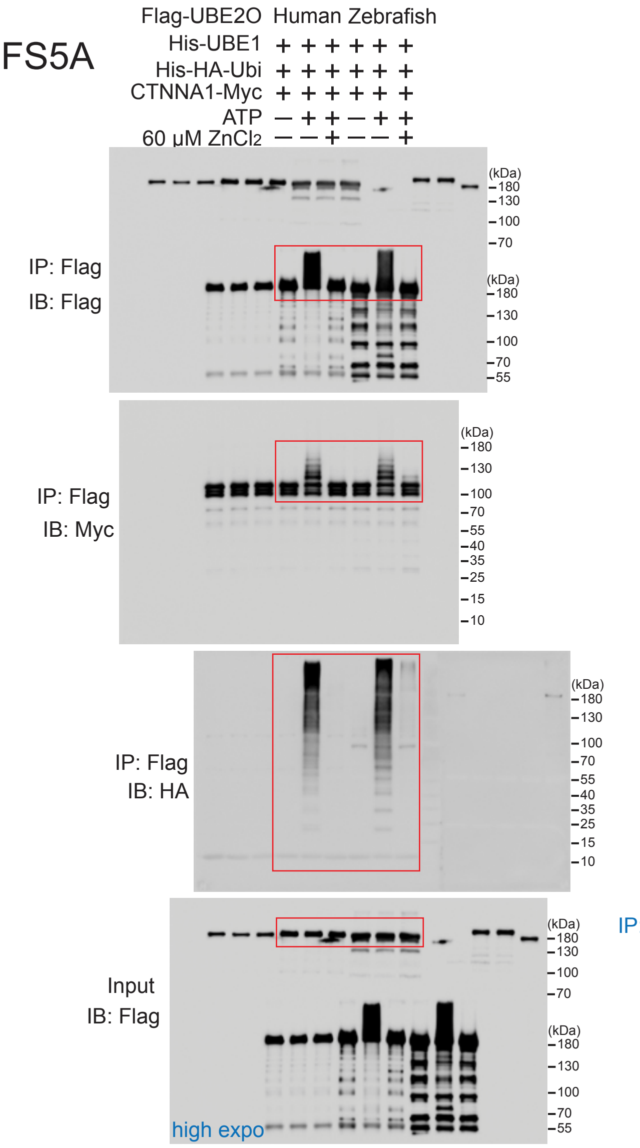

The input and IP samples were resolved in separate SDS-PAGE gels, then transferred onto the same nitrocellulose membrane for subsequent anti-Flag immunoblotting

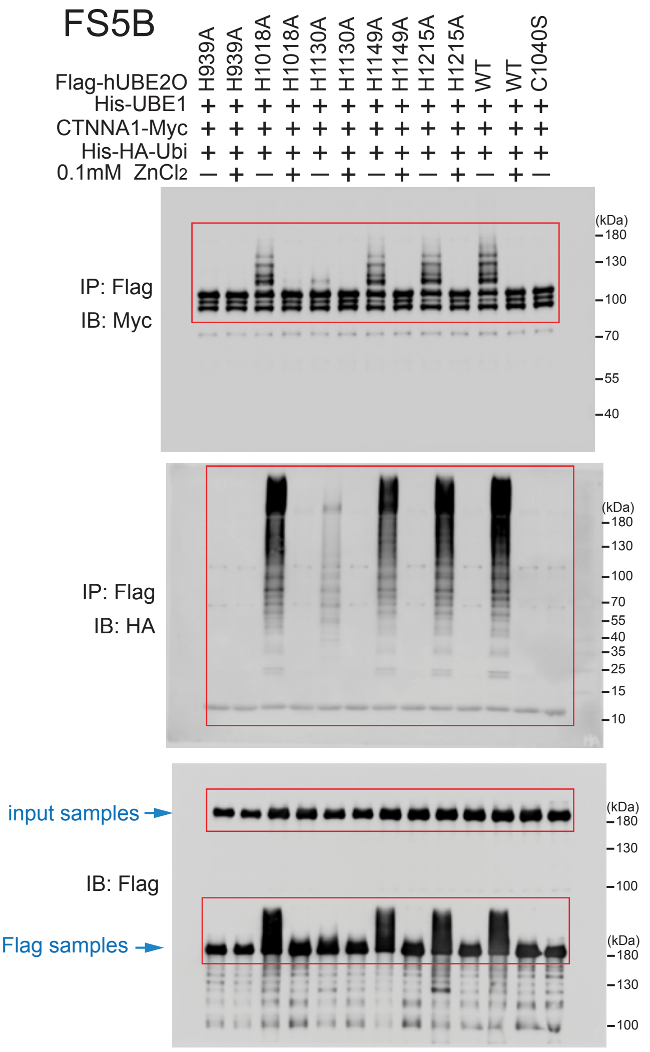

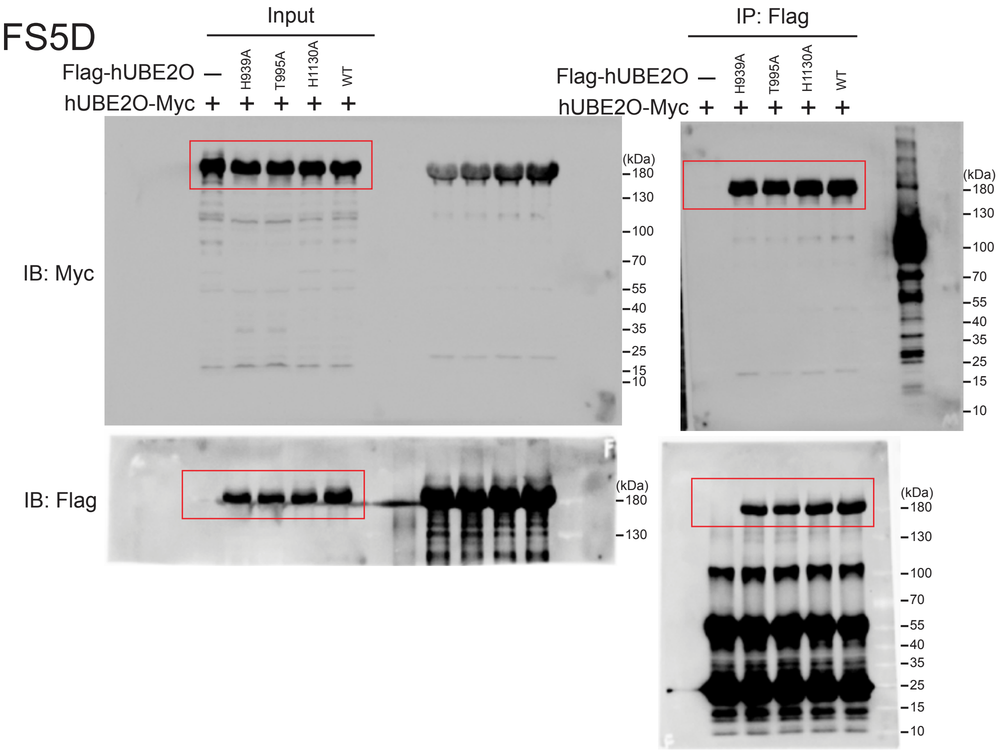

Supplement: Supplementary Material 2 [file mmc2.pdf]
