# Supplementary material for: Allosteric zinc inhibition and interdomain regulation govern the catalytic mechanism of the E3-independent ubiquitin-conjugating enzyme hUBE2O
Source: J Biol Chem. 2025 Dec 30;302(2):111122. doi: 10.1016/j.jbc.2025.111122 (PMC12835413; doi:10.1016/j.jbc.2025.111122)
Supplement: Supplementary Material 3 [file mmc3.pdf]

Supplemental Figures for

Allosteric zinc inhibition and interdomain regulation govern the catalytic mechanism of the E3-independent ubiquitin-conjugating enzyme hUBE2O

Dan Xiang, Xiaoxiao Tang, Ruona Shi, Shuqi Dong and Xiaofei Zhang*

*Correspondence: zhang_xiaofei@gibh.ac.cn

This PDF file includes:

Figure S1. C1040 is the sole catalytic cysteine of hUBE2O. 3

Figure S2. Self-ubiquitination barely regulates hUBE2O activity. 5

Figure S3. hUBE2O's activity is resistant to phosphorylation. 6

Figure S4. Zinc ions, but not phosphorylation inhibits the enzymatic activity of hUBE2O. 8

Figure S5. Zinc ions coordinate with specific cysteine residues in hUBE2O. 10

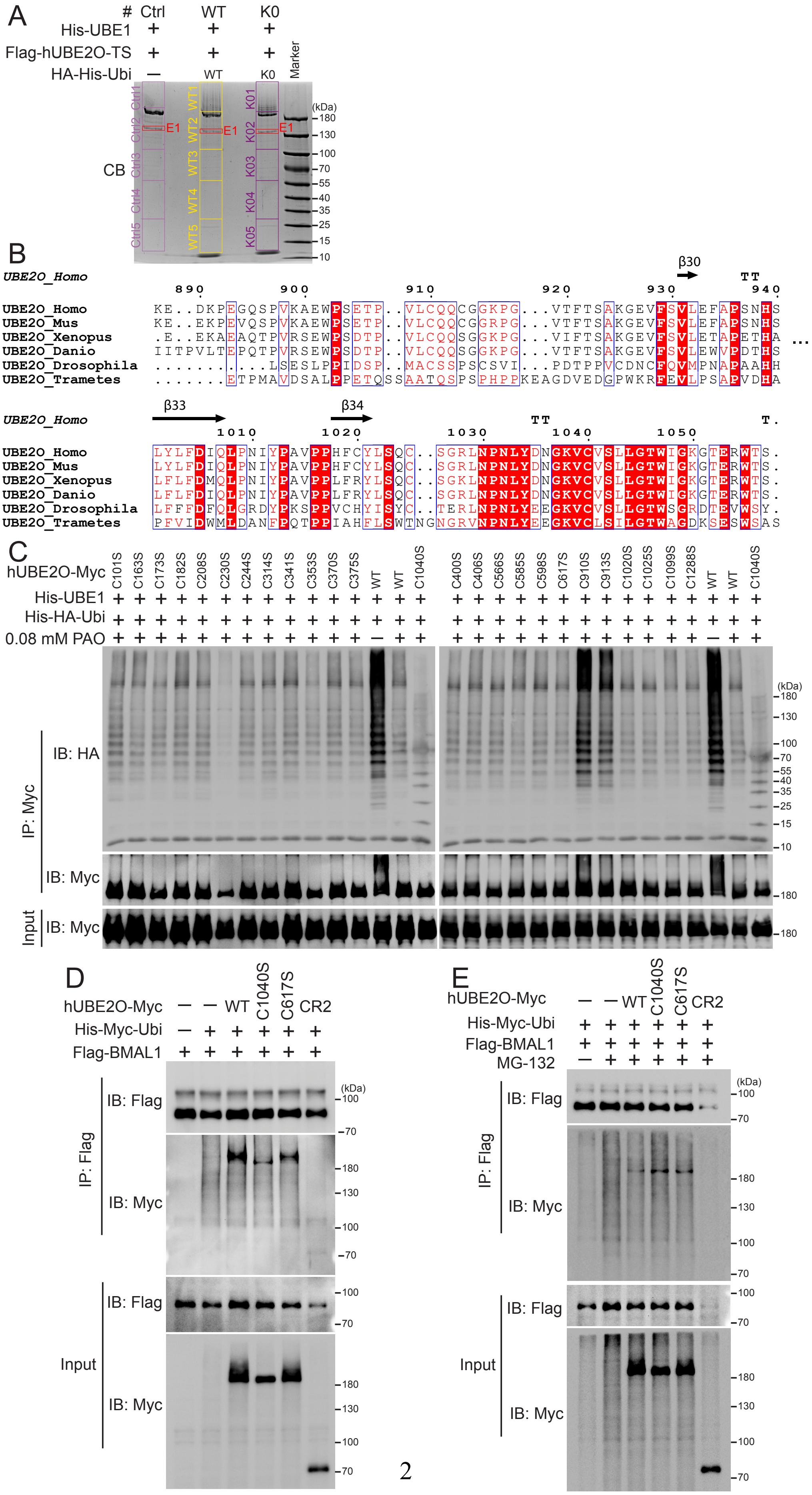
A
Ctrl WT K0
His-UBE1 + + +
Flag-hUBE2O-TS + + +
HA-His-Ubi — WT K0
Marker
CB
Ctrl1 Ctrl2 Ctrl3 Ctrl4 Ctrl5
WT1 WT2 WT3 WT4 WT5
K01 K02 K03 K04 K05
E1
(kDa)
180 130 100 70 55 40 35 25 15 10
B
UBE2O_Homo
β30
TT
890 900 910 920 930 940
UBE2O_Homo KE..DKPEGQSPVKAEWPSETP..VLCQQCGGKPG...VTFTSAKGEVFSVLEFAPSNHS
UBE2O_Mus KE..EKPEVQSPVKAEWPSETP..VLCQQCGGRPG...VTFTSAKGEVFSVLEFAPSNHS
UBE2O_Xenopus .E..EKAEAQTPVRSEWPSDTP..VLCQQSGGKPG...VIFTSAKGEVFSVLETAPETHA
UBE2O_Danio IITPVLTEPQTPVRSEWPSDTP..VLCQQSGGKPG...VTFTSAKGEVFSVLEWVPDTHS
UBE2O_Drosophila ...........LSESLPPIDSP..MACSSPSCSVI...PDTPPVCDNCFQVMPNAPAAHH
UBE2O_Trametes .......ETPMAVDSALPPETQSSAATQSPSPHPPKEAGDVEDGPWKRFEVLPSAPVDHA
...
UBE2O_Homo
β33 β34
TT T.
1010 1020 1030 1040 1050
UBE2O_Homo LYLFDIQLPNIYPAVPPHFCYLSQC..SGRLNPNLYDNGKVCVSLLGTWIGKGTERWTS.
UBE2O_Mus LYLFDIQLPNIYPAVPPHFCYLSQC..SGRLNPNLYDNGKVCVSLLGTWIGKGTERWTS.
UBE2O_Xenopus LFLFDMQLPNIYPAVPPLFRYLSQC..SGKLNPNLYDNGKVCVSLLGTWIGKGTERWTS.
UBE2O_Danio LFLFDIQLPNIYPAVPPLFRYLSQC..SGRLNPNLYDNGKVCVSLLGTWIGKGTERWTS.
UBE2O_Drosophila LFFFDFQLGRDYPKSPPVCHYISYC..TERLNPNLYEEGKVCVSLLGTWSGRDTEVWSY.
UBE2O_Trametes PFVIDWMLDANFPQTPPIAHFLSWTNGNGRVNPNLYEEGKVCLSILGTWAGDKSESWSAS
C
hUBE2O-Myc C101S C163S C173S C182S C208S C230S C244S C314S C341S C353S C370S C375S WT WT C1040S C400S C406S C566S C585S C598S C617S C910S C913S C1020S C1025S C1099S C1288S WT WT C1040S
His-UBE1
His-HA-Ubi
0.08 mM PAO
IB: HA
IP: Myc
IB: Myc
Input
IB: Myc
(kDa)
180 130 100 70 55 40 35 25 15 10
180
180
D
hUBE2O-Myc — — WT C1040S C617S CR2
His-Myc-Ubi — + + + + +
Flag-BMAL1 + + + + + +
IP: Flag
IB: Flag
IB: Myc
Input
IB: Flag
IB: Myc
(kDa)
100 70 180 130 100 70
E
hUBE2O-Myc — — WT C1040S C617S CR2
His-Myc-Ubi + + + + + +
Flag-BMAL1 + + + + + +
MG-132 — + + + + +
IP: Flag
IB: Flag
IB: Myc
Input
IB: Flag
IB: Myc
(kDa)
100 70 180 130 100 70

Figure S1. C1040 is the sole catalytic cysteine of hUBE2O.

A, Coomassie Blue staining of the indicated ubiquitination reactions separated by SDS–PAGE. The indicated bands (excluding E1) were excised and subjected to in-gel tryptic digestion followed by mass spectrometry analysis. *B,* multiple sequence alignment of UBE2Os across different species. Identical residues are colored in red and specially highlighted with red frames; similar residues across the group are highlighted in blue boxes and colored in red, with exceptions in black. *C,* immunoblots show C910S and C913S mutations are resistant to PAO-mediated inhibition among all 25 hUBE2O cysteine to serine mutations. *D* and *E*, ubiquitination assays in HEK293T cells show neither full-length hUBE2O nor C617 mutant catalyze detectable BMAL1 ubiquitination with (*E*) or without (*D*) 12 h treatment of 10 mM MG-132 under our experimental conditions. Experiments were repeated at least twice, one representative result is shown. Source data for this figure are available in supporting information as: SourceDataFS1.

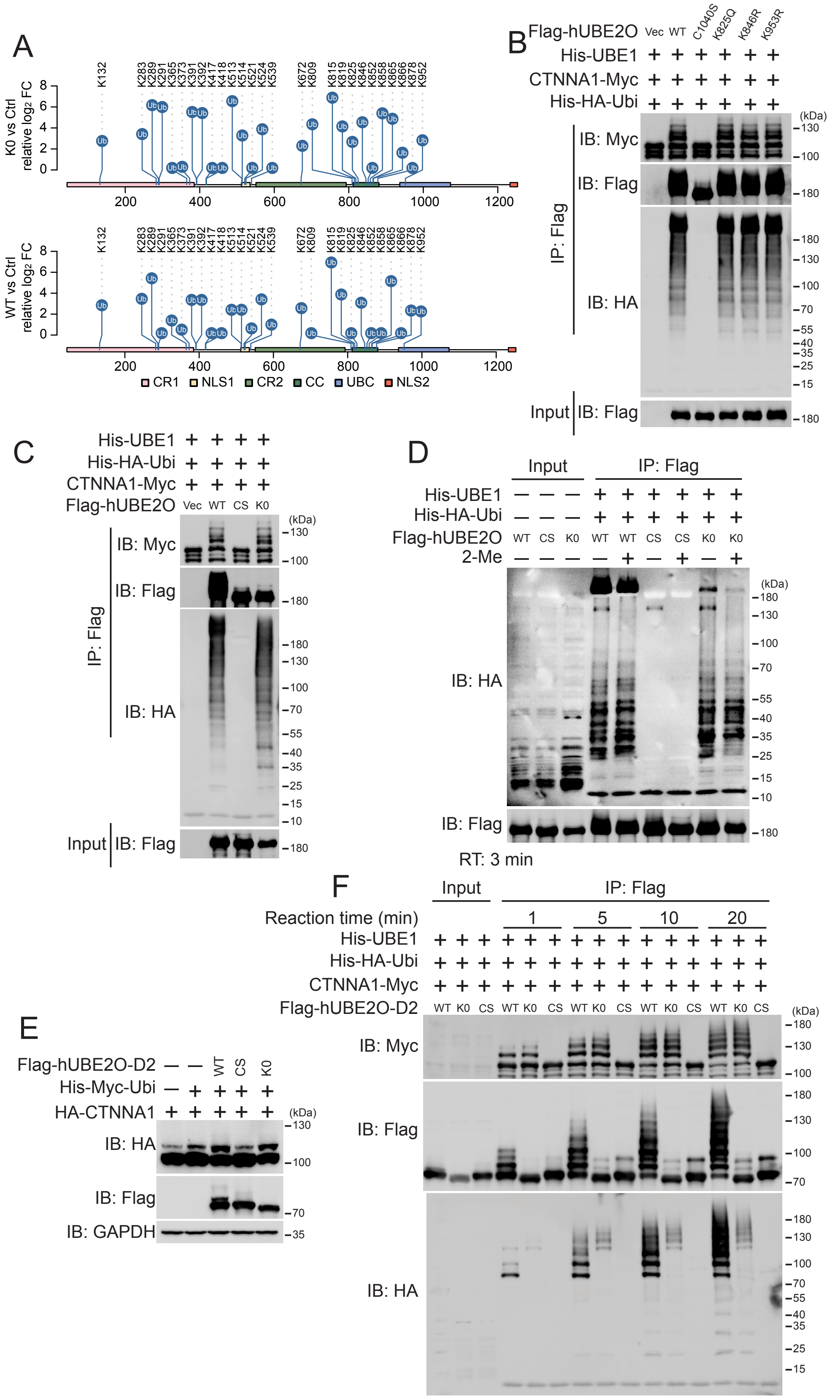
A
K0 vs Ctrl relative log2 FC
WT vs Ctrl relative log2 FC
K132 K283 K289 K291 K365 K373 K391 K392 K417 K418 K513 K514 K521 K524 K539 K672 K809 K815 K819 K825 K846 K852 K858 K865 K866 K878 K952
Ub
0 2 4 6 8
200 400 600 800 1000 1200
CR1 NLS1 CR2 CC UBC NLS2
B
Flag-hUBE2O Vec WT C1040S K825Q K846R K953R
His-UBE1
CTNNA1-Myc
His-HA-Ubi
IP: Flag
IB: Myc
IB: Flag
IB: HA
Input IB: Flag
(kDa)
C
His-UBE1
His-HA-Ubi
CTNNA1-Myc
Flag-hUBE2O Vec WT CS K0
IP: Flag
IB: Myc
IB: Flag
IB: HA
Input IB: Flag
(kDa)
D
Input
IP: Flag
His-UBE1
His-HA-Ubi
Flag-hUBE2O WT CS K0 WT WT CS CS K0 K0
2-Me
IB: HA
IB: Flag
(kDa)
RT: 3 min
E
Flag-hUBE2O-D2 WT CS K0
His-Myc-Ubi
HA-CTNNA1
IB: HA
IB: Flag
IB: GAPDH
(kDa)
F
Input
IP: Flag
Reaction time (min) 1 5 10 20
His-UBE1
His-HA-Ubi
CTNNA1-Myc
Flag-hUBE2O-D2 WT K0 CS
IB: Myc
IB: Flag
IB: HA
(kDa)

Figure S2. Self-ubiquitination barely regulates hUBE2O activity.

A, schematic presentation of the location of hUBE2O self-ubiquitinylated sites detected by mass spectrometry. Detailed data are available in datasets S1. K0: lysine-null. *B,* in vitro ubiquitination assay shows the self-ubiquitination (IB: Flag), polyubiquitin chain formation (IB: HA) and CTNNA1 ubiquitination catalyzing (IB: Myc) ability of the indicated hUBE2O WT, C1040S and KR mutations expressed in HEK293T cells. *C,* in vitro ubiquitination assay shows the self-ubiquitination (IB: Flag), polyubiquitin chain formation (IB: HA) and CTNNA1 ubiquitination catalyzing (IB: Myc) abilities of the indicated hUBE2O WT, C1040S (CS) and lysine-null (K0) mutations expressed in HEK293T cells. *D,* E2~Ubiquitin adducts formation assay shows the E2~Ubiquitin adducts and polyubiquitin chain formation abilities of the indicated hUBE2O WT, C1040S (CS) and lysine-null (K0) mutations expressed in HEK293T cells. *E*, immunoblots show that ablation of self-ubiquitination sites on hUBE2O-D2 barely affects hUBE2O-D2-mediated CTNNA1 ubiquitination in HEK293T cells. HEK293T cells were transfected with the indicated plasmids for 40 h. Cells were harvested for western blot analyses. *F*, immunoblots show the in vitro time-course enzymatic activity of the indicated hUBE2O-D2 variants (WT, C1040S, and lysine-null K0) expressed in HEK293T cells. All experiments except for mass spectrometry identification were repeated at least twice, one representative result is shown. Source data for this figure are available in supporting information as: SourceDataFS2.

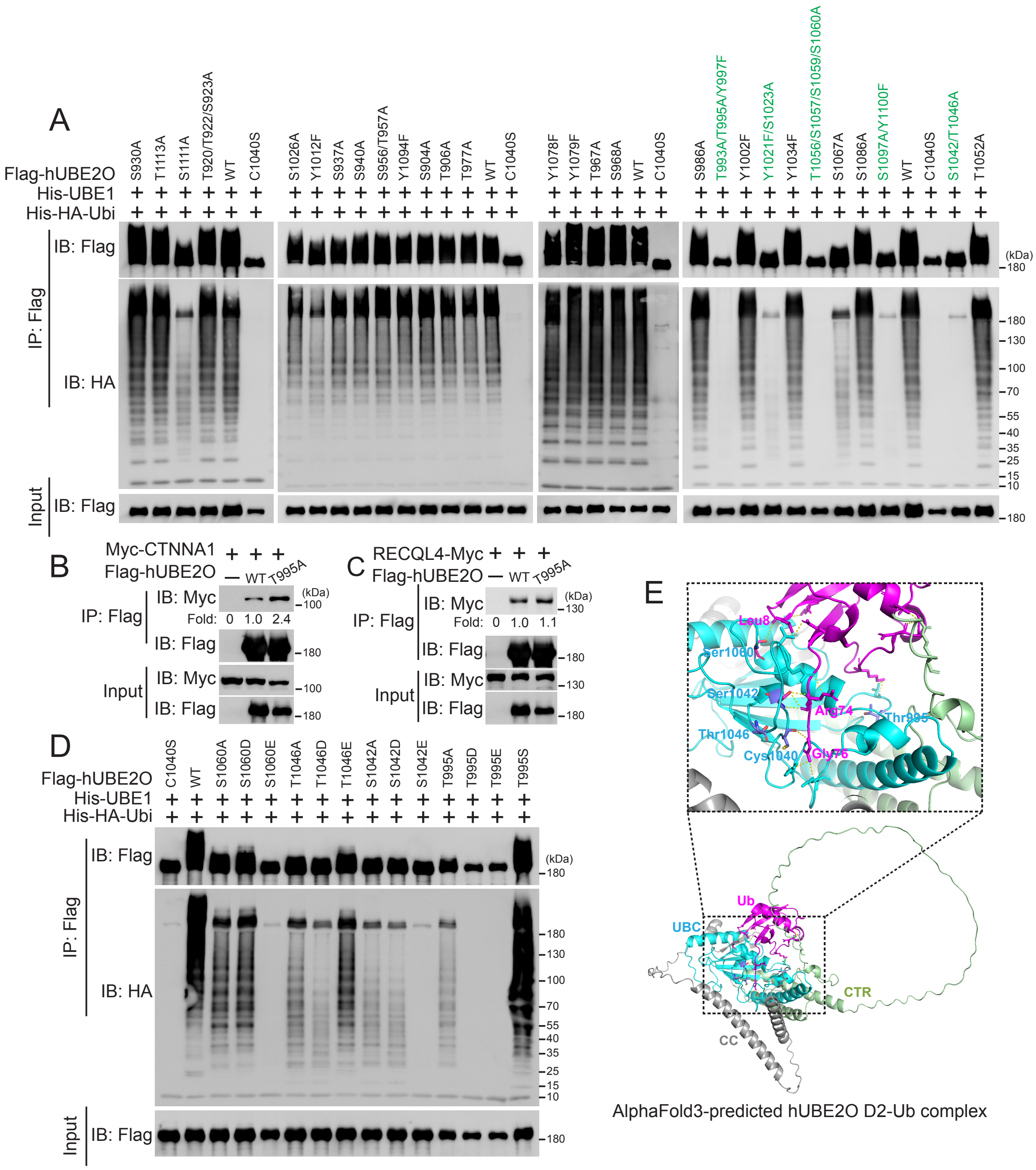

Figure S3. hUBE2O's activity is resistant to phosphorylation.

A, in vitro ubiquitination assay shows the self-ubiquitination (IB: Flag) and polyubiquitin chain formation (IB: HA) abilities of the HEK293T-expressed hUBE2O WT, C1040S and mutations of indicated serine, threonine and tyrosine residues in the UBC domain. *B* and *C,* co-immunoprecipitation assays show the T995A mutant remains its interaction with CTNNA1 and RECQL4. *D*, in vitro ubiquitination assay shows the self-ubiquitination (IB: Flag) and polyubiquitin chain formation (IB: HA) abilities of the HEK293T-expressed hUBE2O WT, C1040S and the phosphorylation-prevented or phosphorylation-mimetic mutations of S1060, T1046, S1042 and T995 residues. *E,* a structural model of hUBE2O-D2 (801-1292aa) in complex with Ubiquitin generated using AlphaFold3. hUBE2O is shown as cartoon representation with individual domains labeled and color coded. The CC domain of hUBE2O (gray), UBC domain of hUBE2O (cyan), the CTR domain of hUBE2O (green), and Ubiquitin

(magenta) are shown as cartoons (bottom panel). The inter-domain interaction residues of hUBE2O are shown as cyan sticks (S1060, T1046, S1042, C1040 and T995 residues are highlighted as slate sticks), nitrogen and oxygen atoms are respectively in blue and red, and the corresponding interaction residues of Ubiquitin are shown as magenta sticks. All experiments were repeated at least twice, one representative result is shown. Source data for this figure are available in supporting information as: SourceDataFS3.

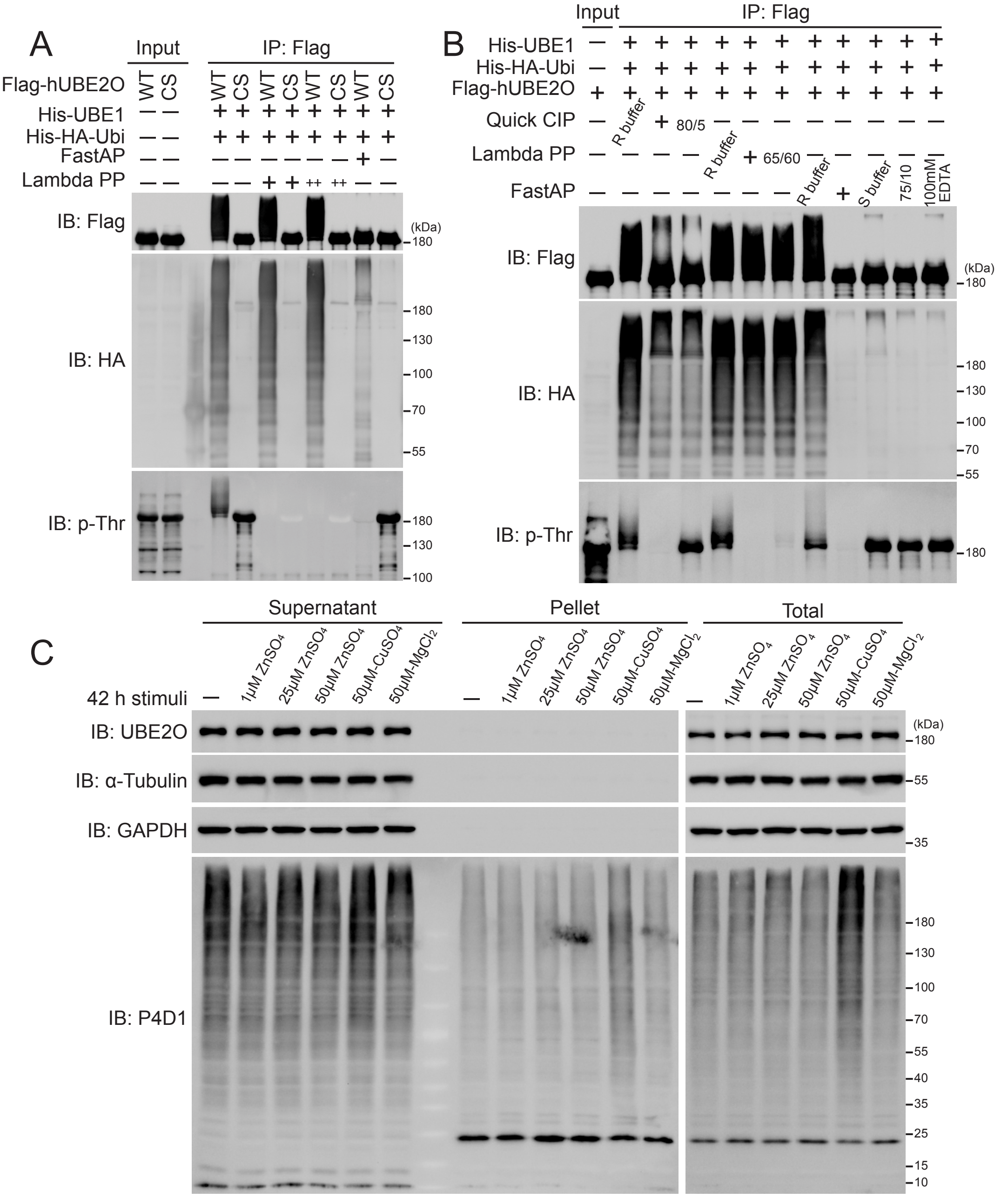

Figure S4. Zinc ions, but not phosphorylation inhibits the enzymatic activity of hUBE2O.

A, in vitro dephosphorylation followed by ubiquitination assay shows Lambda PP and FastAP treatment successfully reduce threonine phosphorylation of hUBE2O (IB: p-Thr) but only FastAP treatment completely abolishes hUBE2O's self-ubiquitination (IB: Flag). ++ indicates 2 ×Lambda PP phosphatase was used. *B,* in vitro dephosphorylation followed by ubiquitination assay shows the storage buffer of FastAP, but not dephosphorylation abolishes hUBE2O's self-ubiquitination. Quick CIP, Lambda PP and FastAP were inactivated by incubating at 80°C for 5 min (80/5), 65°C for 60 min (65/60) and 75°C for 10 min (75/10), respectively. FastAP was also inactivated by 100 mM EDTA before dephosphorylation assay. *C,* immunoblots show zinc ions barely affect endogenous UBE2O solubility/accumulation or overall polyubiquitination patterns in HEK293T cells. HEK293T cells treated with the indicated metal ions for 48 h were collected for western blot analysis according to the experimental procedures. P4D1 antibody

was used to detect ubiquitin, polyubiquitin and ubiquitinated proteins. All experiments were repeated at least twice, one representative result is shown. Source data for this figure are available in supporting information as: SourceDataFS4.

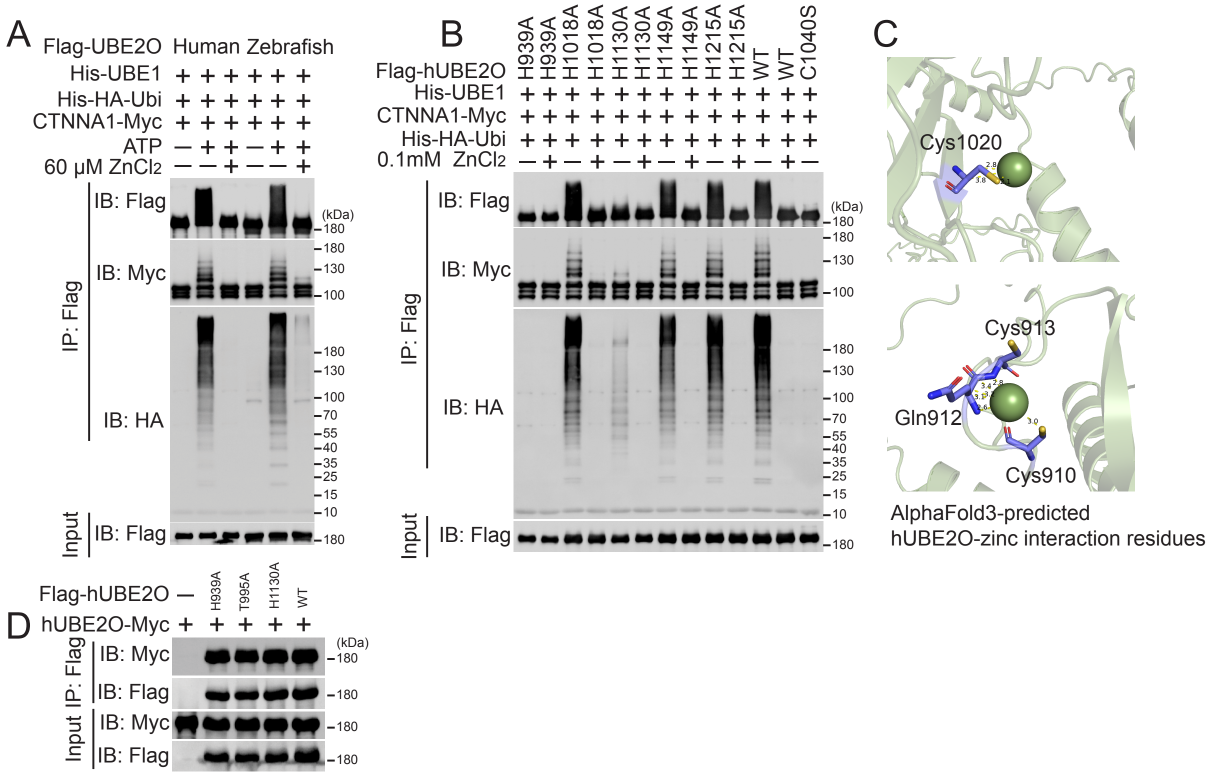

Figure S5. Zinc ions coordinate with specific cysteine residues in hUBE2O.

A and *B,* in vitro ubiquitination assay shows the self-ubiquitination (IB: Flag), polyubiquitin chain formation (IB: HA) and CTNNA1 ubiquitination catalyzing (IB: Myc) abilities of the indicated hUBE2O, its zebrafish homolog and histidine to alanine mutations expressed in HEK293T cells. *C,* structural models of the interaction residues of hUBE2O to zinc ions in the hUBE2O-6zinc complex generated using AlphaFold3. hUBE2O is shown as green cartoon. The interaction residues of hUBE2O are shown as slate sticks, nitrogen, oxygen and sulfur atoms are respectively in blue, red and yellow. *D,* immunoprecipitation assay shows the interactions between hUBE2O WT and the H939A, T995A or H1130A mutations. All experiments were repeated at least twice, one representative result is shown. Source data for this figure are available in supporting information as: SourceDataFS5.
