# Supplementary material for: Allosteric zinc inhibition and interdomain regulation govern the catalytic mechanism of the E3-independent ubiquitin-conjugating enzyme hUBE2O
Source: J Biol Chem. 2025 Dec 30;302(2):111122. doi: 10.1016/j.jbc.2025.111122 (PMC12835413; doi:10.1016/j.jbc.2025.111122)
Supplement: Supplementary Material 4 [file mmc4.docx]

## Supplemental Table S1. Oligos used in this study

| Name | Sequence |
| --- | --- |
| UBE2O-C101S-F | TCGGGGTCCTCCGAGGCCGGGGGCGCGGGCCACGA |
| UBE2O-C101S-R | CTCGGAGGACCCCGAGCTCCCGCGGCCCTCCTCCT |
| UBE2O-C163S-F | AGTCAGTCTGGCACGGTGATCGACGTCAACATCGACTG |
| UBE2O-C163S-R | CGTGCCAGACTGACTGTCGGTGGATCGCATGTGC |
| UBE2O-C173S-F | ATCGACTCTGCCGTCAAGCTCATCGGCACCAACTG |
| UBE2O-C173S-R | GACGGCAGAGTCGATGTTGACGTCGATCACCGTGC |
| UBE2O-C182S-F | CCATCATCTATCCCGTCAACAGCAAG |
| UBE2O-C182S-R | AGTTGGTGCCGATGAGCTTGAC |
| UBE2O-C208S-F | CCTGGCTGGGGAAGGTCTACGAC |
| UBE2O-C208S-R | AGTCATAGGCAATGTAGTCCCCA |
| UBE2O-C230S-F | CCTCCATGAACACGGAAGATGGCG |
| UBE2O-C230S-R | ACCTGGCGCCGTTGGATAGC |
| UBE2O-C244S-F | CCCCGCACGTCAGCGACTCGG |
| UBE2O-C244S-R | AGACGTCGTAGAGCTTGGCGC |
| UBE2O-C314S-F | CTCCAGGGGGCACGGACAGCG |
| UBE2O-C314S-R | AGAAACTCTTGGTAATCCATGTAACTT |
| UBE2O-C341S-F | CTCGGATCCTTTGACCATGCTCAGCGGCAGCTTG |
| UBE2O-C341S-R | GTCAAAGGATCCGAGACGCTTCACCCTGCCTAGG |
| UBE2O-C353S-F | CTCTGTATGTCTTCCCAGCCAAGGTAGAGCC |
| UBE2O-C353S-R | AGCGCTCCCCAAGCTGCCGC |
| UBE2O-C370S-F | CTCCAGAAAAAAACTGCGCCCAGG |
| UBE2O-C370S-R | ATTCCCAGGCAATCTTGGCTGG |
| UBE2O-C375S-F | CCGCCCAGGGGGAGGGCTCTAT |
| UBE2O-C375S-R | AGTTTTTTTCTGGACATTCCCAGGCAAT |
| UBE2O-C400S-F | CCTCCCCAGACACCCAGTGTTCC |
| UBE2O-C400S-R | ATGACATGATCCGCACAACCTGC |
| UBE2O-C406S-F | ACCCAGTCTTCCCGGGACCATTCCATGGAAGACCCAGAC |
| UBE2O-C406S-R | CCGGGAAGACTGGGTGTCTGGGGAGCATGACATGATCC |
| UBE2O-C566S-F | CCAACATCCGCTCCAACGA |
| UBE2O-C566S-R | ATTCCACGGAGCCATCCTG |
| UBE2O-C585S-F | TTCTCCCCTGGAGACTTCGTG |
| UBE2O-C585S-R | CTCGTTGTTGTCCAGGTGGTG |
| UBE2O-C598S-F | CTCCAGACCCTGCTGTCTACG |
| UBE2O-C598S-R | AGCTCTGGACTCGCTTATCTACC |
| UBE2O-C617S-F | TCCATGGTGAAGTGGTTCAAGCT |
| UBE2O-C617S-R | GGTACGGCCGATGTGGTCC |
| UBE2O-C910S-F | CCCAGCAGTGTGGCGGCAAGCC |
| UBE2O-C910S-R | ACAGCACCGGGGTTTCGCTGG |
| UBE2O-C913S-F | CTGGCGGCAAGCCTGGCGTCAC |
| Name | Sequence |
| UBE2O-C913S-R | ACTGCTGGCACAGCACCGGGG |
| UBE2O-C1020S-F | GCCCCCCCACTTCTCCTAC |
| UBE2O-C1020S-R | ACGGCTGGGTAGATGTTGGG |
| UBE2O-C1025S-F | TCCAGTGGCCGCCTGAAC |
| UBE2O-C1025S-R | TTGGGAGAGGTAGCAGAAGTGGG |
| UBE2O-C1099S-F | AGTCGCTCTTACAATGAGATGGCGCTGATCCGCG |
| UBE2O-C1099S-R | ATTGTAAGAGCGACTGTTTTCATAGCCTTCCTGCAGGC |
| UBE2O-C1288S-F | CCACAGAGGACAAGATCGAACAAAAACT |
| UBE2O-C1288S-R | ACTCCGGCATGCCTGCCTCTA |
| UBE2O-F | ATGGCGGATCCCGCAGCCCCCA |
| UBE2O-R | CTTGTCCTCTGTGCACTCCGGCA |
| UBE2O-A427-F | GCGGAGTCTGCCAGCCCTGAG |
| UBE2O-K801-F | AAGGCTGGCAAGGACGGGCCAC |
| UBE2O-E901-F | GAGTGGCCCAGCGAAACCCCG |
| UBE2O-L1115-R | CAGCTGGGTCATGGACTGCACCAC |
| UBE2O-T828-R | AGTCATGTTCTTGAGGCTCTCCAGG |
| UBE2O-V928-F | GTCTTCTCCGTACTGGAGTTTGCAC |
| UBE2O-C1288-R | GCACTCCGGCATGCCTGCC |
| UBE2O-E1147-R | TTCCAGCCAGGACTCGATACGG |
| UBE2O-P1246-F | CCTGAGAAGAGTGGCTACCCTG |
| hBMAL1-F | ATGGCAGACCAGAGAATGGACAT |
| hBMAL1-R | CAGCGGCCATGGCAAGTCACT |
| S1026A-F | CCAATGCGCTGGCCGCCTGAACCCCAACCTGTATGAC |
| S1026A-R | GCGGCCAGCGCATTGGGAGAGGTAGCAGAAGTGGGG |
| S1042/T1046A-F | TGGGCTTCTGGATTGGAAAGGGGACAGAGAGG |
| S1042/T1046A-R | GGAGGGCGACACACACCTTCCCATTGTC |
| S1042D-F | GTGTGTCGATCTCCTGGGCACCTGGATTGGAAAGGGG |
| S1042D-R | CCAGGAGATCGACACACACCTTCCCATTGTCATACAGGTTG |
| S1042E-F | GTGTGTCGAACTCCTGGGCACCTGGATTGGAAAGGGG |
| S1042E-R | CCAGGAGTTCGACACACACCTTCCCATTGTCATACAGGTTG |
| S1060D-F | CAAGTCCGATCTTCTCCAGGTGCTCATCTCCATCCAAGG |
| S1060D-R | GGAGAAGATCGGACTTGCTTGTCCACCTCTCTGTCCCC |
| S1060E-F | CAAGTCCGAACTTCTCCAGGTGCTCATCTCCATCCAAGG |
| S1060E-R | GGAGAAGTTCGGACTTGCTTGTCCACCTCTCTGTCCCC |
| S1067A-F | GCCATCCAAGGTCTGATCCTGGTAAATG |
| S1067A-R | GATGAGCACCTGGAGAAGGCTGGAC |
| S1086A-F | GCTGACCGAGGCCTGCAGGAAG |
| S1086A-R | GTCGAAGCCGGCTTCGTTGTAG |
| S1097A/Y1100F-F | TGTTTCAATGAGATGGCGCTGATCCG |
| S1097A/Y1100F-R | GCGAGCGTTTTCATAGCCTTCCTGCAGGC |
| S1111A-F | GCCATGACCCAGCTGGTGCGG |
| Name | Sequence |
| S1111A-R | CTGCACCACGCGGATCAGCG |
| S904A-F | GTGGCCCGCCGAAACCCCGGTGCTGTGCCAGCAGTG |
| S904A-R | GGTTTCGGCGGGCCACTCAGCCTTCACAGGTGACTGC |
| S930A-F | GCCGTACTGGAGTTTGCACCC |
| S930A-R | GAAGACCTCGCCCTTGGCG |
| S937A-F | GCAAATCATTCTTTTAAGAAAATTGAGTTCCAG |
| S937A-R | GGGTGCAAACTCCAGTACGG |
| S940A-F | GCTTTTAAGAAAATTGAGTTCCAGCCTCC |
| S940A-R | ATGATTTGAGGGTGCAAACTCCAG |
| S956/T957A-F | GCAGTGCGGAAGGAGATGGCGCTGCTG |
| S956/T957A-R | GGCGAAGAACTTCTTGGCTTCTGGAGGCTG |
| S968A-F | GGCTACCGCACTGCCTGAGGGCATCATGGTCAAGAC |
| S968A-R | GGCAGTGCGGTAGCCAGCAGCGCCATCTCCTTCCG |
| S986A-F | GCAGCTCTCATCAAGGGCCCCAC |
| S986A-R | GAAGAGGTCCATTCTATCTTCAAAAGTC |
| SingeS1060A-F | GCCCTTCTCCAGGTGCTCATCTCC |
| SingeS1060A-R | GGACTTGCTTGTCCACCTCTCTG |
| SingleS1023A-F | CTACCTCGCCCAATGCAGTGGCCGCCTGAACCCCA |
| SingleS1023A-R | CATTGGGCGAGGTAGCAGAAGTGGGGGGGCACGGC |
| SingleS1042A-F | GTGTGTCGCCCTCCTGGGCACCTGGATTGGAAAGGGG |
| SingleS1042A-R | CAGGAGGGCGACACACACCTTCCCATTGTCATACAGGTTGG |
| SingleS1057A-F | GTGGACAGCCAAGTCCAGCCTTCTCCAGGTGCTCATC |
| SingleS1057A-R | GGACTTGGCTGTCCACCTCTCTGTCCCCTTTCCAATCCAG |
| SingleS1059A-F | AAGCAAGGCCAGCCTTCTCCAGGTGCTCATCTCCA |
| SingleS1059A-R | AGGCTGGCCTTGCTTGTCCACCTCTCTGTCCCCTT |
| SingleS1097A-F | TGAAAACGCTCGCTGTTACAATGAGATGGCGCTGATCCG |
| SingleS1097A-R | ACAGCGAGCGTTTTCATAGCCTTCCTGCAGGCCTCG |
| SingleT1046A-F | CCTGGGCGCCTGGATTGGAAAGGGGACAGAGAGGTG |
| SingleT1046A-R | TCCAGGCGCCCAGGAGGCTGACACACACCTTCCC |
| SingleT1056A-F | GAGGTGGGCAAGCAAGTCCAGCCTTCTCCAGGTGC |
| SingleT1056A-R | TTGCTTGCCCACCTCTCTGTCCCCTTTCCAATCCAGG |
| SingleT993A-F | GGGCCCCGCTCGAACCCCCTACGAGGATGGCCTCTAC |
| SingleT993A-R | GTTCGAGCGGGGCCCTTGATGAGAGCTGAGAAGAGGTCC |
| SingleT995A-F | CACTCGAGCCCCCTACGAGGATGGCCTCTACTTGTTTG |
| SingleT995A-R | TAGGGGGCTCGAGTGGGGCCCTTGATGAGAGCTGA |
| SingleY1021F-F | TTCTGCTTCCTCTCCCAATGCAGTGGCCGCCTGAA |
| SingleY1021F-R | GGAGAGGAAGCAGAAGTGGGGGGGCACGGCTGGGT |
| SingleY1100F-F | CGCTGTTTCAATGAGATGGCGCTGATCCGCGTGGT |
| SingleY1100F-R | CTCATTGAAACAGCGACTGTTTTCATAGCCTTCCTGCAGG |
| SingleY997F-F | ACCCCCTTCGAGGATGGCCTCTACTTGTTTGACATCCAG |
| SingleY997F-R | ATCCTCGAAGGGGGTTCGAGTGGGGCCCTTGATGA |
| Name | Sequence |
| T1046D-F | CCTGGGCGATTGGATTGGAAAGGGGACAGAGAGGTGGAC |
| T1046D-R | CAATCCAATCGCCCAGGAGGCTGACACACACCTTCCC |
| T1046E-F | CCTGGGCGAATGGATTGGAAAGGGGACAGAGAGGTGGAC |
| T1046E-R | CAATCCATTCGCCCAGGAGGCTGACACACACCTTCCC |
| T1052A-F | AAAGGGGGCAGAGAGGTGGACAAGCAAGTCCAGCC |
| T1052A-R | CTCTCTGCCCCCTTTCCAATCCAGGTGCCCAGGAG |
| T1056/S1057/S1059/S1060A-F | AGGCCGCCCTTCTCCAGGTGCTCATCTCCATC |
| T1056/S1057/S1059/S1060A-R | TGGCTGCCCACCTCTCTGTCCCCTTTCC |
| T1113A-F | GCCCAGCTGGTGCGGCGGCCCC |
| T1113A-R | CATGGACTGCACCACGCGGATCAGC |
| T906A-F | CAGCGAAGCCCCGGTGCTGTGCCAGCAGTGTGGCG |
| T906A-R | ACCGGGGCTTCGCTGGGCCACTCAGCCTTCACAGG |
| T920/T922/S923A-F | CCGCCGCCAAGGGCGAGGTCTTCTC |
| T920/T922/S923A-R | CGAAGGCGACGCCAGGCTTGCCGCCAC |
| T967A-F | GCTGGCTGCCTCACTGCCTGAGGGCATCATGGTCA |
| T967A-R | AGTGAGGCAGCCAGCAGCGCCATCTCCTTCCGCAC |
| T977A-F | GGTCAAGGCTTTTGAAGATAGAATGGACCTCTTCTCAGCTCTC |
| T977A-R | TCAAAAGCCTTGACCATGATGCCCTCAGGCAGTGAG |
| T993A/T995A/Y997F-F | CCCCTTCGAGGATGGCCTCTACTTGTTTG |
| T993A/T995A/Y997F-R | GCTCGAGCGGGGCCCTTGATGAGAGCTG |
| T995D-F | CACTCGAGATCCCTACGAGGATGGCCTCTACTTGTTTGAC |
| T995D-R | CGTAGGGATCTCGAGTGGGGCCCTTGATGAGAGCTG |
| T995E-F | CACTCGAGAACCCTACGAGGATGGCCTCTACTTGTTTGAC |
| T995E-R | CGTAGGGTTCTCGAGTGGGGCCCTTGATGAGAGCTG |
| T995S-F | CACTCGATCTCCCTACGAGGATGGCCTCTACTTGTTTGAC |
| T995S-R | CGTAGGGAGATCGAGTGGGGCCCTTGATGAGAGCTGA |
| Y1002F-F | TCTTGTTTGACATCCAGCTCCCC |
| Y1002F-R | AGAGGCCATCCTCGTAGGGGG |
| Y1012F-F | AACATCTTCCCAGCCGTGCCCCCCCACTTCTGCTAC |
| Y1012F-R | GGCTGGGAAGATGTTGGGGAGCTGGATGTCAAACAAGTAG |
| Y1021F/S1023A-F | CGCCCAATGCAGTGGCCGCCTGAAC |
| Y1021F/S1023A-R | AGGAAGCAGAAGTGGGGGGGCACG |
| Y1034F-F | TTGACAATGGGAAGGTGTGTGTCAG |
| Y1034F-R | ACAGGTTGGGGTTCAGGCGG |
| Y1078F-F | GAACCATTCTACAACGAAGCCGGCTTCGACAGTGAC |
| Y1078F-R | GTTGTAGAATGGTTCATTTACCAGGATCAGACCTTGG |
| Y1079F-F | CCATACTTCAACGAAGCCGGCTTCGACAGTGACCG |
| Y1079F-R | TTCGTTGAAGTATGGTTCATTTACCAGGATCAGACCTTG |
| Y1094F-F | GAAGGCTTTGAAAACAGTCGCTGTTACAATGAGATGGCG |
| Y1094F-R | GTTTTCAAAGCCTTCCTGCAGGCCTCGGTCACTGTC |
| H939A-F | CTCAAATGCTTCTTTTAAGAAAATTGAGTTCCAGCC |
| Name | Sequence |
| H939A-R | TAAAAGAAGCATTTGAGGGTGCAAACTCCAGTACGGA |
| H1018A-F | GCCCCCCGCTTTCTGCTACCTCTCCCAATGCAGTGGC |
| H1018A-R | AGCAGAAAGCGGGGGGCACGGCTGGGTAGATGTTGGG |
| H1130A-F | CAGGCAAGCTTTTAGCACTGGTGGCTGGCGGCTGGTG |
| H1130A-R | TGCTAAAAGCTTGCCTGATCTCCTGCTCAAAGACCTCGG |
| H1149A-F | GGAAACCGCTGCCCTGCTGGAGAAGGCCCAGGCACT |
| H1149A-R | CAGGGCAGCGGTTTCCAGCCAGGACTCGATACGGTTC |
| H1215A-F | CAGGGACGCTACAGACCAGACTTCGGAGACCGCACCAG |
| H1215A-R | GGTCTGTAGCGTCCCTGCTAGCTGAGGCCAGGCCCTG |

Supplemental Table S1 lists oligos used in this study.
